# The molecular genealogy of sequential overlapping inversions implies both homologous chromosomes of a heterokaryotype in an inversion origin

**DOI:** 10.1038/s41598-019-53582-8

**Published:** 2019-11-18

**Authors:** Dorcas J. Orengo, Eva Puerma, Unai Cereijo, Montserrat Aguadé

**Affiliations:** 1 Departament de Genètica, Microbiologia i Estadística, Facultat de Biologia, i Institut de Recerca de la Biodiversitat (IRBio), Universitat de Barcelona, Barcelona, Spain; 2 Present Address: Centre for Research in Agricultural Genomics, CSIC-IRTA-UAB-UB, Campus UAB, Bellaterra (Cerdanyola del Vallès), 08193 Barcelona, Spain

**Keywords:** Evolution, Genetics

## Abstract

Cytological and molecular studies have revealed that inversion chromosomal polymorphism is widespread across taxa and that inversions are among the most common structural changes fixed between species. Two major mechanisms have been proposed for the origin of inversions considering that breaks occur at either repetitive or non-homologous sequences. While inversions originating through the first mechanism might have a multiple origin, those originating through the latter mechanism would have a unique origin. Variation at regions flanking inversion breakpoints can be informative on the origin and history of inversions given the reduced recombination in heterokaryotypes. Here, we have analyzed nucleotide variation at a fragment flanking the most centromere-proximal shared breakpoint of several sequential overlapping inversions of the E chromosome of *Drosophila subobscura* —inversions E_1_, E_2_, E_9_ and E_3_. The molecular genealogy inferred from variation at this shared fragment does not exhibit the branching pattern expected according to the sequential origin of inversions. The detected discordance between the molecular and cytological genealogies has led us to consider a novel possibility for the origin of an inversion, and more specifically that one of these inversions originated on a heterokaryotype for chromosomal arrangements. Based on this premise, we propose three new models for inversions origin.

## Introduction

Chromosomal inversions are structural rearrangements with chromosomal segments in inverted orientation relative to non-inverted chromosomes. Their presence was first inferred in Drosophila almost a century ago from their genetic effect as suppressors of recombination when in heterozygosis^1^. They could be later observed at the cytological level in the larval salivary glands of *Drosophila melanogaster* as their cells present giant (polytene) chromosomes that exhibit somatic pairing^2^. Cytological methods propelled the study of inversion chromosomal polymorphism in natural populations of several Diptera species across the twentieth century^3–5^. The later molecular characterization of polymorphic inversions breakpoints allowed the identification of inversions by specific PCR amplification of their breakpoints^6–8^. Moreover, bioinformatics methods have been developed to identify inversions by comparing genomes of the same species (*e. g*.^9^) as well as of different species (*e. g*.^10,11^). The different methodological approaches have uncovered the pervasive existence of inversion chromosomal polymorphism across taxa (*i.e*., from bacteria to humans^3,12–18^). Moreover, comparisons between closely related species have revealed that at this level, inversions are the most common structural changes^10,19–21^ and that they play an important role in speciation^22^.

The extensive cytological studies of inversion polymorphism in *Drosophila pseudoobscura* and *D. persimilis* uncovered a series of chromosomal arrangements that differed by overlapping inversions, with similar studies in *D. subobscura* and other species also revealing such series in various chromosomes (as reviewed in^3^). These observations led to the establishment of chromosomal phylogenies under the generally accepted assumption that each inversion had a unique origin. However, the discovery of transposable elements raised the possibility that an inversion could originate repeatedly^23^. Inversions can originate through ectopic recombination between two transposable elements, or other repetitive sequences, present in opposite orientation on the same chromosome. Moreover, inversions can also result from erroneously joining the ends of two breaks on the same chromosome, simply through a cut-and-paste procedure but also from staggered breaks and their subsequent repair^10,24^. These mechanisms are often referred to, respectively, as NAHR —for non-allelic homologous recombination— and NHEJ —for non-homologous end joining. While a certain inversion resulting from NAHR could occur repeatedly^23,25^, the occurrence of any inversion resulting from NHEJ should be a unique event in the history of the species^10,24^. The molecular characterization of polymorphic inversions breakpoints in both *D. melanogaster*^6,9,26^ and *D. subobscura*^27–32^ has revealed that in these species the latter mechanism is prevalent, and therefore that most of their inversions have a unique origin. In contrast, the recent characterization of polymorphic inversions breakpoints in human populations has revealed that many of them are comparatively rather short and originated repeatedly by ectopic recombination between repetitive sequences^33^.

For inversions generated by the NHEJ mechanism, the extreme bottleneck due to their unique origin implies that they are originally devoid of variation. Variation in the inverted region accrues through time as a result of new mutations. An inversion can also acquire variation through recombination —by either double crossovers or gene conversion— with non-inverted chromosomes. The size of the inversion affects this exchange of variation as recombination is highly suppressed near the breakpoint regions of the inverted fragment and it increases with distance to the breakpoints^34^. Variation at regions closer to the breakpoints or flanking the breakpoints themselves might therefore better reflect the history of large inversions like those detected in different Drosophila species.

**Figure 1:**
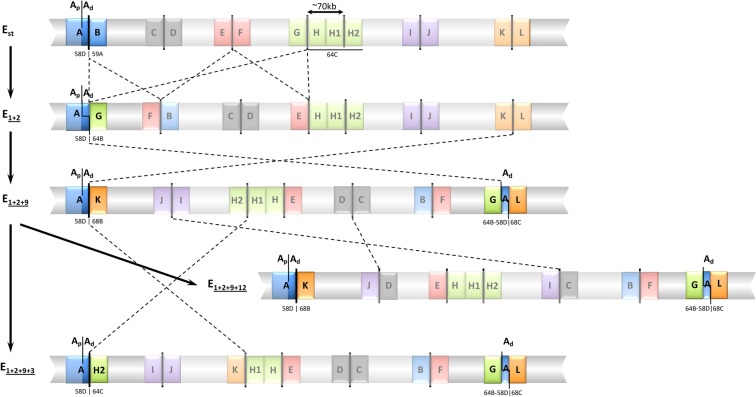
Schematic representation of chromosomal arrangements E_st_, E_1+2_, E_1+2+9_, E_1+2+9+3_, and E_1+2+9+12_ of *Drosophila subobscura*. Inversions originating these arrangements are indicated by crossed lines. Breakpoint regions including the A fragment that is shared by various inversions are highlighted. The cytological location of these breakpoints on the Kunze-Mühl and Müller^36^ map is given. A_p_ and A_d_ refer, respectively, to the proximal and distal section of the A fragment (see text). The A_d_ section was duplicated when inversion E_9_ originated. Not at scale.

**Figure 2:**
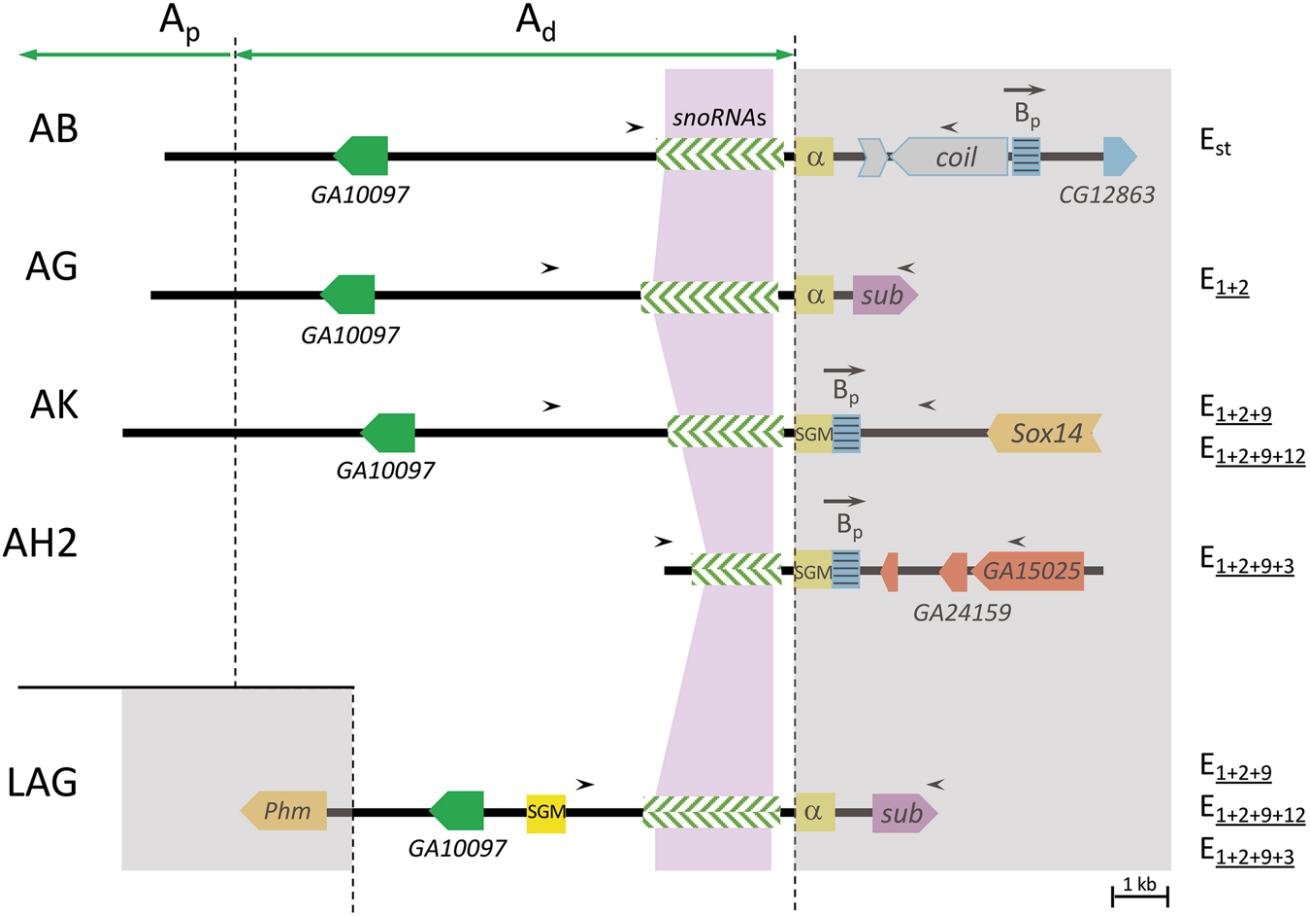
Functional annotation of breakpoint regions spanning fragment A. Annotation extracted from our previous work^28,32^. Breakpoint regions are named as in Fig. 1 except for the GAL region that is here presented in reverse orientation (and therefore named LAG) to facilitate its comparison with the other regions. Chromosomal arrangements presenting each breakpoint region are indicated on the rightmost part of the figure. A_p_ and A_d_ refer, respectively, to the proximal and distal sections of the A part (see text). Small black arrowheads indicate the location of amplification primers. The sequenced A fragment at each breakpoint is rose shadowed. B_p_ refers to the proximal fragment of the ancestral B part of the E_st_ arrangement with the arrow indicating its orientation relative to the breakpoint. Colored arrowed boxes represent protein-coding regions. Green-striped boxes represent the presence of multiple snoRNA genes. SGM indicates an SGM element. α indicates an alpha element exhibiting some similarity to the SGM element.

*D. subobscura* exhibits a rich inversion polymorphism that affects its five acrocentric chromosomes. Each of these chromosomes presents overlapping inversions that occur sequentially leading in some cases to chromosomal complexes formed by three or more extant chromosomal arrangements. The E chromosome (or Muller C element) stands out in this sense because it harbors a complex formed by the successive accumulation of inversions on the ancestral chromosome, hereafter referred as E_st_. Even if the E_1+2+9_ complex consists in eight different chromosomal arrangements^35^ formed through nine different inversions *—*E_1_, E_2_, E_9_, E_3_, E_4_, E_5_, E_12_, E_15_ and E_18_— only a subset of them coexist in any particular population. Here we have focused on the subset of these inversions*—*E_1_, E_2_, E_9_, E_3_ and E_12_— generating the most common derived chromosomal arrangements in the Mediterranean area —E_1+2_, E_1+2+9_, E_1+2+9+3_ and E_1+2+9+12_ (Fig. 1). At the cytological level, four of those inversions *—*E_1_, E_2_, E_9_ and E_3_*—* were considered to share their most centromere-proximal breakpoint^36^. Our molecular characterization of their breakpoints revealed that the proximal breakpoint regions of these inversions —*i.e*., regions AB, AG, AK, and AH2 in Fig. 1— share their A part^28,32^. It should be noted that the ~9-kb long fragment of the A part immediately flanking the breakpoint was duplicated when inversion E_9_ originated^32^. This duplicated fragment is therefore present in the here named GAL region of the E_1+2+9_ arrangement and its derivatives E_1+2+9+3_ and E_1+2+9+12_ (Figs 1 and 2). The A part is hereafter subdivided into two sections according to the extent of the duplication: A_p_ and A_d_ for the centromere-proximal and centromere-distal sections, respectively —corresponding the A_d_ section to the duplicated stretch. It should be, moreover, noted that arrangement E_1+2+9+12_ also shares region AK with arrangement E_1+2+9_, as the E_12_ inversion did not affect the E_9_ inversion breakpoints (Fig. 1).

In the present work, we have estimated the level of nucleotide variation in the ~2-kb long segment of the A part closest to the breakpoint shared by inversions E_1_, E_2_, E_9_ and E_3_ (hereafter named fragment A; Fig. 2) as variation in fragment A of chromosomal arrangements E_st_, E_1+2_, E_1+2+9_, E_1+2+9+3_ and E_1+2+9+12_ can be informative on the origin and history of these inversions.

## Results and Discussion

### Nucleotide variation at a fragment flanking multiple inversion breakpoints

Twenty-nine heterokaryotypic individuals from a wild population sampled on the outskirts of Barcelona in 2014^8^ were used to separately obtain the nucleotide sequence of the A fragment from each homologous E chromosome whenever possible. Supplementary Table S1 shows these individuals karyotypes and also the breakpoint regions including fragment A —AB, AG, AK, AH2 and GAL (Fig. 1)— that were PCR amplified in each individual. Figure 2 presents the functional annotation of these breakpoint regions^28,32^ as well as the size of the amplified fragments spanning fragment A.

The 29 sampled individuals are expected to harbor 80 A fragments given the presence of the GAL region in E_1+2+9_, E_1+2+9+3_ and E_1+2+9+12_ chromosomes. Only 50 of these fragments were successfully amplified, sequenced and analyzed. Concerning amplification, the AG region —exclusive of the E_1+2_ arrangement— could only be independently amplified in E_st_/E_1+2_ heterokaryotypic individuals given that all other heterokaryotypes exhibit two copies corresponding to the AG and GAL regions (Supplementary Table S1). Moreover, some difficulties were encountered to completely sequence the A fragment of some amplified regions due to the presence of long thymidine (T) runs in the AG and GAL regions and the presence of inserted SGM (Subobscura Guanche Madeirensis) transposable elements^37^ in the AK region of various individuals. These characteristics and the presence of a series of small and similar snoRNA genes in fragment A increased the difficulty to perform multiple alignments of this fragment sequences, which required some manual curation. Supplementary Table S2 shows the nucleotide polymorphisms detected in the 50 sequenced A fragments.

Regions immediately flanking an inversion breakpoint are good markers of the inversion history as their variation in inverted chromosomes is mainly due to new mutations. Indeed, only gene conversion would contribute to the acquisition of variation from non-inverted chromosomes as double-crossover events are negligible in these regions. Variation at the A fragment of breakpoint regions AB, AG and AH2 will thus be considered to reflect variation at chromosomal arrangements E_st_, E_1+2_ and E_1+2+9+3_, respectively, whereas that of region AK would reflect variation at arrangements E_1+2+9_ and E_1+2+9+12_, and that of region GAL variation at arrangements E_1+2+9_, E_1+2+9+3_ and E_1+2+9+12_ (Figs 1 and 2). As the AK and GAL regions are present in both E_1+2+9_ and E_1+2+9+12_ chromosomal arrangements and even though the E_12_ inversion did not affect the E_9_ inversion breakpoints (Fig. 1), we tested for any putative differentiation of the AK and GAL sequenced regions between these chromosomal arrangements prior to analyzing variation in this fragment. The estimated *F*_*ST*_ value^38^ for each the AK and GAL regions —0.066 and 0.031, respectively— did not significantly differ from 0 as revealed by the corresponding permutation test (*P* = 0.18 and *P* = 0.31, respectively). Sequences from both arrangements (E_1+2+9_ and E_1+2+9+12_) were therefore grouped for subsequent analyses (hereafter referred to as E_[1+2+9]_).

Table 1 summarizes the analysis of nucleotide polymorphism and divergence —using the complete deletion option— at fragment A from each of the five different breakpoint regions considered (AB, AG, AK, GAL and AH2) and also when jointly considered. Nucleotide diversity at the A fragment varied between the different breakpoint regions, ranging from 0.006 at the AH2 region to 0.015 at the AB region. Similar values were obtained within arrangement when considering the pairwise deletion option (results not shown). The level of nucleotide diversity at fragment A in the different E chromosomal arrangements is of the same order than previously estimated in *D. subobscura* at regions affected by other autosomal and X-linked inversions^39–43^.

**Table 1:** Nucleotide polymorphism and divergence at fragment A from different breakpoint regions.

	Breakpoint region					
	AB	AG	GAL	AK	AH2	Overall
No. samples	18	6	10	11	5	50
No. nucleotides^a^	2003	2168	2305	1275	2118	1143
No. segregating sites (*S*)	104	42	66	56	23	192
No. singletons	23	29	28	29	7	45
No. multiple hit sites	4	0	1	1	0	17
Nucleotide diversity (π)	0.015	0.007	0.010	0.013	0.006	0.031
No. haplotypes (*h*)	17	5	10	11	5	45
Nucleotide divergence (*K*)^b^	0.092	0.088	0.092	0.076	0.096	0.075

^a^After excluding sites with alignment gaps. Some more sites were excluded to estimate *K* due to additional alignment gaps. ^b^With Jukes and Cantor correction. *D. guanche* was used as outgroup.

Variation at fragment A within the different chromosomal arrangements (*i.e*., at the AB, AG, AK and AH2 breakpoint regions) did not consistently increase with their relative age as inferred from the sequential occurrence of inversions E_1_, E_2_, E_9_ and E_3_ (Table 1 and Fig. 1). However, age is not the only aspect that can affect the level of variation at fragment A from the different arrangements. Indeed, its variation could also have been affected by i) the frequency attained by each arrangement, and ii) the putative recent fixation of an adaptive point or structural mutation in any of the arrangements.

### Genetic differentiation between the A fragments of the different breakpoint regions

**Figure 3:**
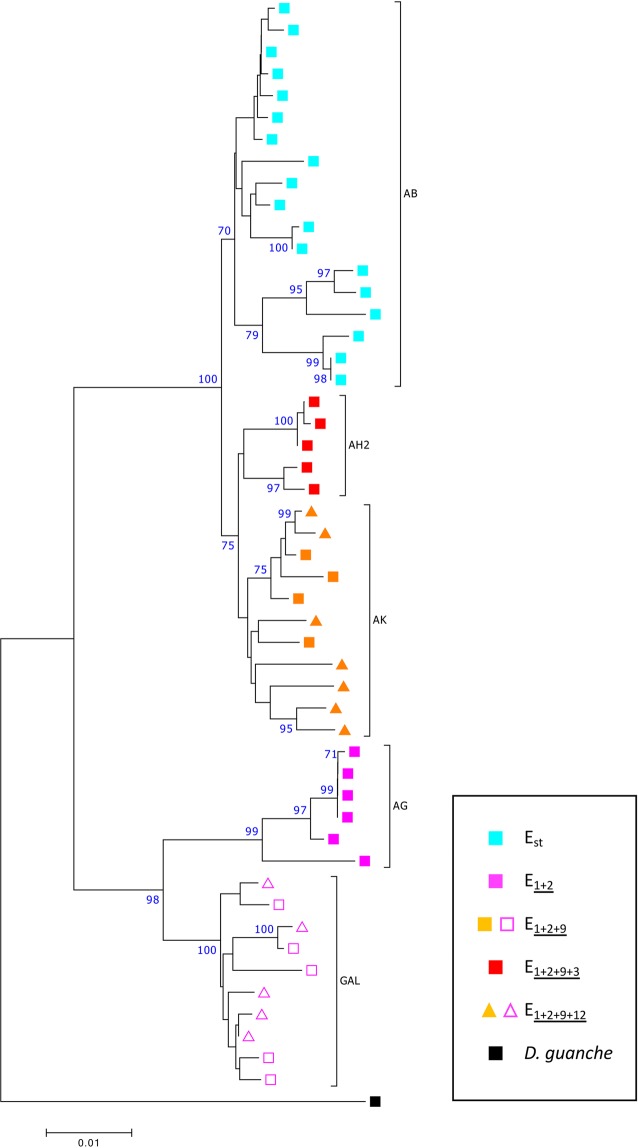
Neighbor-joining tree of the A fragment sequences corresponding to the breakpoint regions of different E chromosomal arrangements. Bootstrap values >70% (based on 1000 replicates) are shown on the tree. Positions with over 5% alignment gaps, missing data, or ambiguous bases were not considered. *D. guanche* was used as outgroup.

Table 2 summarizes the level of genetic differentiation at fragment A between the different breakpoint regions (AB, AG, AK, AH2 and GAL; Fig. 1) as measured by the *F*_ST_ statistic^38^. As expected from the relatively recent origin of the E_1+2+9+3_ chromosomal arrangement through the E_3_ inversion on an E_1+2+9_ chromosome (Fig. 1), the lowest *F*_ST_ estimate for the A fragment is that between the AK and AH2 breakpoint regions. However, in contrast to expectations from the sequential occurrence of inversion E_9_ on an E_1+2_ chromosome and of inversion E_3_ on an E_1+2+9_ chromosome (Fig. 1), the *F*_ST_ estimates for the A fragment were much lower between the AB breakpoint region and both the AK and AH2 breakpoint regions than between the AG breakpoint region and both the AK and AH2 breakpoint regions. This discordant result is clearly reflected in the genealogy inferred from variation at the A fragment, which is based on the 50 A fragment sequences of *D. subobscura* using the *D. guanche* sequence as outgroup (Fig. 3). Sequences from each particular breakpoint region of *D. subobscura* cluster together into differentiated clades. As expected from the cytological phylogeny^44^, sequences from regions AK and AH2 that correspond to the youngest arrangements —E_[1+2+9]_ and E_1+2+9+3_— cluster together. Surprisingly, these sequences group together with the AB sequences that correspond to the oldest arrangement E_st_ and not with the AG sequences that correspond to the E_1+2_ arrangement from which the E_[1+2+9]_ and E_1+2+9+3_ arrangements are considered to be derived. In contrast, sequences from the GAL region corresponding to the E_1+2+9_ arrangement and its derivatives cluster together with the AG sequences, as expected from the sequential occurrence of inversions (Fig. 1).

**Table 2:** Genetic differentiation between the different breakpoint regions.

	AB	AG	GAL	AK	AH2
AB	—	0.0000	0.0000	0.0000	0.0000
AG	0.8026	—	0.0000	0.0003	0.0017
GAL	0.7555	0.6937	—	0.0000	0.0003
AK	0.3498	0.7880	0.73374	—	0.0006
AH2	0.4532	0.8429	0.80161	0.23662	—

*F*_*ST*_ estimates between each pair of chromosomal regions are shown in the lower part of the matrix and the corresponding *P*-values obtained from 10000 permutations in its upper part.

The discordance between the molecular genealogy inferred from variation at the A fragment that flanks the proximal breakpoint of four sequentially originated inversions and their cytology-based phylogeny led us to check two possible sources of this being an artifactual result: i) sequence misalignment, and ii) putative bias in the sequenced sample. Concerning the first possible source, we checked again the multiple alignment of fragment A. No progress was made in this sense as the alignment had already been manually curated. Concerning the putative biased sampling of sequences from the natural population that might be associated to considering only heterokaryotypic individuals, comparison of the frequencies of the five chromosomal arrangements in the 29 sequenced heterokaryotypic individuals and in the complete sample from 2014^8^ revealed no significant difference (G test = 5.195; d. f. = 4; P = 0.214). Moreover, the genealogy inferred from the A fragment sequences obtained from the homokaryotypic lines used to identify and characterize the different inversions breakpoint regions^28,32^ exhibits the same branching pattern than that inferred from the heterokaryotypic individuals (Supplementary Fig. S1).

### Inversion E_9_ originated in an inversion heterokaryotype

The overlapping character of inversions E_1_, E_2_, E_9_ and E_3_ implies their sequential occurrence, which is reflected in their cytology-based phylogeny (Fig. 1). The molecular genealogy inferred from variation at the shared A fragment of regions AB, AG, AK and AH2 would be expected to exhibit the same branching pattern than the cytology-based phylogeny given that this fragment immediately flanks the most centromere-proximal breakpoint of the corresponding inversions. Nevertheless, the molecular genealogy does not conform to afore mentioned expectations. The detected discordance —*i.e*., the clustering of arrangements E_[1+2+9]_ and E_1+2+9+3_ with E_st_ instead of with E_1+2_, as inferred from the A fragment sequences (Fig. 3 and Supplementary Fig. S1)— would place the focus on the origin of inversion E_9_.

A clue to understand the detected discordance between the molecular genealogy and the cytology-based phylogeny stems from the comparison of the extended AB, AG, AK and AH2 sequences (Fig. 2) from the homokaryotypic lines^28,32^. This comparison revealed that the AK and AH2 sequences share an ~500-nt long fragment adjacent to the distal end of section A_d_ (hereafter named fragment B_p_; Fig. 2). In E_1+2_ chromosomes, fragment B_p_ is absent from their AG region and present in the B part of their BF region (Fig. 1). Moreover, fragment B_p_ is present in the B part of the AB region of E_st_ chromosomes even if at a different position (Fig. 2). In order to ascertain whether fragment B_p_ was a repetitive element, it was used as query for both a RepeatMasker (http:// www.repeatmasker.org/) search, and a BLAST search against the *D. guanche* genome^45^. The negative result of the first search and the reduced number of very partial hits returned by the second search would not yield any support for it being a transposable element or any other repetitive sequence that could have been replaced since the E_9_ inversion originated. These observations clearly indicate that inversion E_9_ would have captured its B_p_ fragment from an E_st_ chromosome (Fig. 2) when originating. Taking into account that the B part of E_st_ chromosomes (Fig. 1) suffered several structural changes prior and after the E_1+2_ arrangement originated^28^, it can be inferred that the B_p_ fragment was ancestrally at a proximal position relative to the A fragment of E_st_. These results and the detected discordance between the molecular genealogy and cytology-based phylogeny of the studied arrangements have led us to consider that inversion E_9_ occurred in an individual heterokaryotypic for arrangements E_st_ and E_1+2_. The newly formed E_1+2+9_ chromosome could have, thus, acquired some features of the E_st_ A fragment during the E_9_ inversion process.

### New models to explain the origin of inversion E_9_

The presence in inverted orientation of the ~9-kb long fragment named A_d_ at both inversion E_9_ breakpoints had been considered a clear signal that this fragment was duplicated when inversion E_9_ originated^32^. Two previous NHEJ models had been proposed to explain the presence at both breakpoints of inverted chromosomes of a duplicated fragment relative to the single copy present in only one of the breakpoints of non-inverted chromosomes^10^. These models are: i) the isochromatid model that considers two staggered breaks in a single chromatid occurring during premeiotic mitosis and ii) the chromatid model that considers two breaks in each of two sister chromatids, occurring during meiotic prophase. Neither of these previously proposed models can account for the detected discordance between the molecular genealogy and cytology-based phylogeny of the studied arrangements because they are both chromatid models. Here, we propose three new chromosome models that would explain the detected discordance under the assumption that inversion E_9_ originated in an individual heterokaryotypic for arrangements E_st_ and E_1+2_.

**Figure 4:**
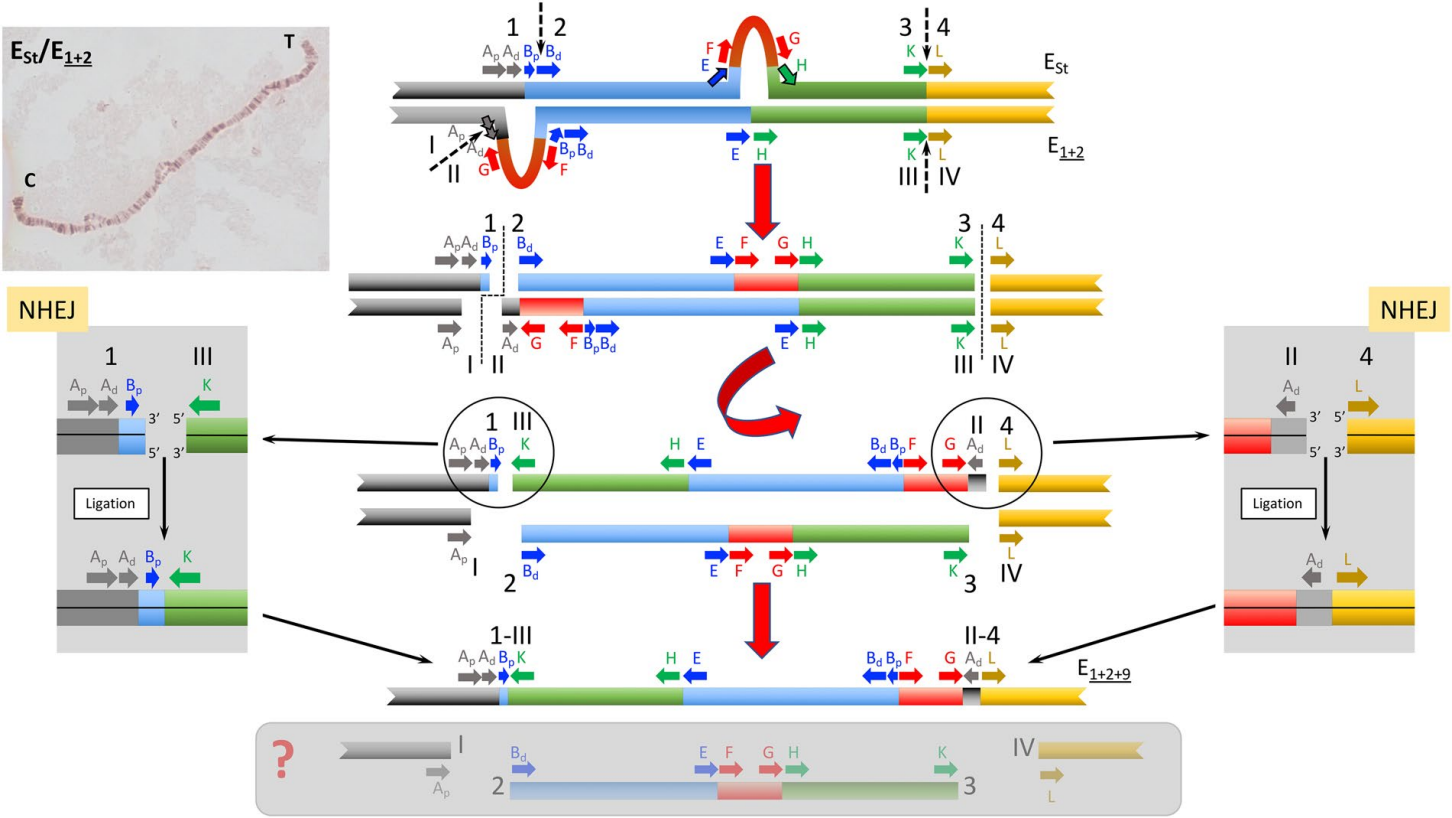
Schematic representation of the NHEJ-4-chromosome model for the origin of inversion E_9_. The sequential steps of how arrangement E_1+2+9_ could have originated from an E_st_/E_1+2_ heterokaryotypic individual through inversion E_9_ are graphically represented in the central part of the figure. Fragments flanking the different breakpoint regions are labeled as in Fig. 1. Initial state: pairing of the E homologous chromosomes of an E_st_/E_1+2_ heterokaryotypic individual with discontinuous arrows indicating the location of future breaks. Parts flanking future breaks are labeled in E_st_ and E_1+2_ homologues by ordinal numbers and roman numerals, respectively. Upper left corner inset, image of an E_st_/E_1+2_ polytene chromosome preparation. First step: a total of four breaks considering both homologous chromosomes, with the two breaks in the proximal region occurring at different sites in both homologues —between sections B_p_ and B_d_ of the E_st_ homologue and between sections A_p_ and A_d_ of the E_1+2_ homologue—, and those in the distal region (KL) occurring at the same site. Discontinuous lines indicate the location of breaks. Second step: inversion of the central fragment of the E_1+2_ homologue and resolution of the double-strand breaks. Insets on both sides of the central scheme highlight the resolution phase. Final state: result of the inversion process with the generation of the E_1+2+9_ arrangement. Also shown within a grey-shaded box are the chromosomal fragments that might have resulted —highlighted by a question mark,?— in an evolutionary unsuccessful arrangement.

The first model proposed —named NHEJ-4-chromosome model— considers that inversion E_9_ originated through the NHEJ mechanism and resulted from four breaks occurring on both homologous chromosomes of a heterokaryotypic individual (Fig. 4). According to this model, both homologous chromosomes —E_st_ and E_1+2_— would have been simultaneously broken at two different sites (staggered break) in the proximal region and at the same site in the distal region. The proximal break on the E_st_ arrangement would have occurred past the B_p_ fragment and that on the E_1+2_ arrangement at the limit between the A_p_ and A_d_ sections. The repair of these chromosomal breaks would have been resolved by the NHEJ mechanism so that the excised central part of the E_1+2_ chromosome would have been rejoined in inverted orientation to the external E_st_ fragments, giving rise to inversion E_9_. The rest of chromosomal fragments could have been joined in different ways or even not have been joined at all, with their putative product/s not having survived to present.

**Figure 5:**
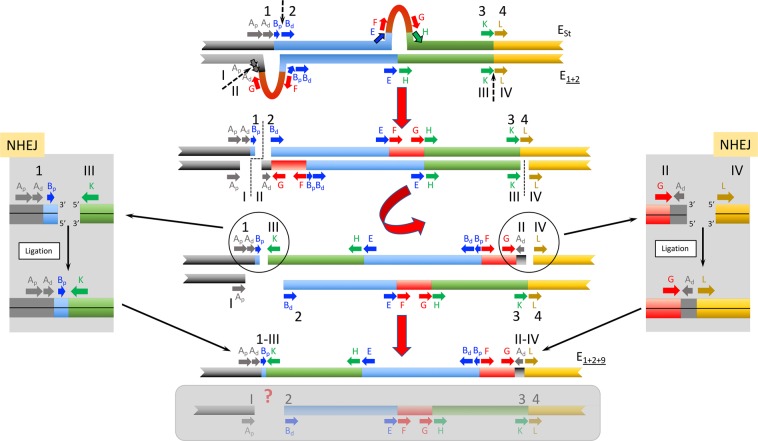
Schematic representation of the NHEJ-3-chromosome model for the origin of inversion E_9_. The sequential steps of how arrangement E_1+2+9_ could have originated from an E_st_/E_1+2_ heterokaryotypic individual through inversion E_9_ are graphically represented in the central part of the figure. Fragments flanking the different breakpoint regions are labeled as in Fig. 1. Initial state: pairing of the E homologous chromosomes of an E_st_/E_1+2_ heterokaryotypic individual with discontinuous arrows indicating the location of future breaks. Parts flanking breakpoints are labeled as in Fig. 4. First step: a total of three breaks considering both homologous chromosomes, with the two breaks in the proximal region occurring at different sites in both homologues —between sections B_p_ and B_d_ of the E_st_ homologue and between sections A_p_ and A_d_ of the E_1+2_ homologue—, and that in the distal region (KL) occurring at the E_1+2_ homologue. Discontinuous lines indicate the location of breaks. Second step: inversion of the central fragment of the E_1+2_ homologue and resolution of the double-strand breaks. Insets on both sides of the central scheme highlight the resolution phase. Final state: result of the inversion process with the generation of the E_1+2+9_ arrangement. Also shown within a grey-shaded box are the chromosomal fragments that might have resulted —highlighted by a question mark,?— in an E_st_ chromosome lacking sections A_d_ and B_p_.

The second model proposed —named NHEJ-3-chromosome model— also considers that inversion E_9_ originated through the NHEJ mechanism, but that it resulted from only three breaks (Fig. 5). According to this model, a staggered break similar to that of the NHEJ-4-chromosome model would have occurred at the proximal region. In contrast, only the E_1+2_ chromosome would have suffered an additional break at the distal region. The repair of these chromosomal breaks would have been resolved by the NHEJ mechanism so that the excised central part of the E_1+2_ chromosome would have been rejoined in inverted orientation to the proximal E_st_ and distal E_1+2_ fragments, respectively, giving rise to inversion E_9_. This model is similar to that proposed by Sharakhov *et al*.^24^ as it also implies three breaks on both homologous chromosomes but it differs from that model in two fundamental aspects: i) the breaks would have been repaired by the NHEJ and not by the NAHR mechanism, and ii) the inversion would have originated in a heterokaryotypic individual.

**Figure 6:**
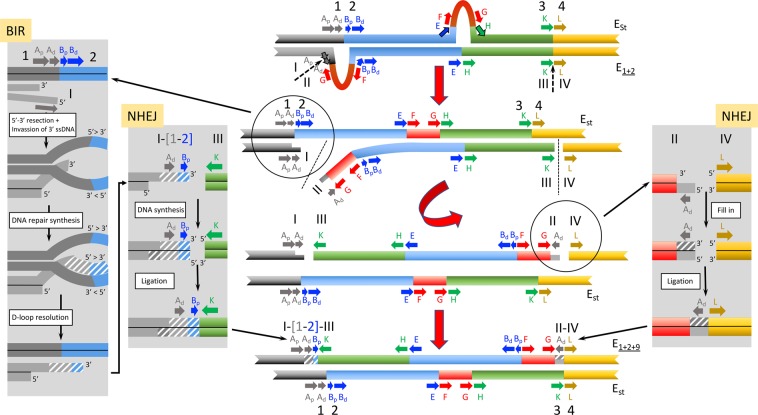
Schematic representation of the BIR-NHEJ-chromosome model for the origin of inversion E_9_. The sequential steps of how arrangement E_1+2+9_ could have originated from an E_st_/E_1+2_ heterokaryotypic individual through inversion E_9_ are graphically represented in the central part of the figure. Fragments flanking the different breakpoint regions are labeled as in Fig. 1. Initial state: pairing of the E homologous chromosomes of an E_st_/E_1+2_ heterokaryotypic individual with discontinuous arrows indicating the location of future breaks. Parts flanking breakpoints are labeled as in Fig. 4. First step: two breaks in the E_1+2_ homologue, with that in the proximal region occurring between sections A_p_ and A_d_, and that in the distal region occurring between the K and L parts of the KL breakpoint region. Discontinuous lines indicate the location of breaks. Second step: inversion of the central fragment of the E_1+2_ homologue and resolution of the double-strand break of the proximal region through the BIR pathway and that of the distal region through the NHEJ mechanism. Insets on both sides of the central scheme highlight the different steps of the BIR and NHEJ pathways, respectively. Final state: result of the inversion process with the generation of the E_1+2+9_ arrangement. Also shown is the E_st_ chromosome that did not undergo any break.

The third model proposed —named BIR-NHEJ-chromosome model— considers that inversion E_9_ originated through two breaks on a single sister chromatid of the E_1+2_ homologous chromosome of an E_st_/E_1+2_ heterokaryotypic individual (Fig. 6). The proximal break would have been repaired through the Break-Induced Replication (BIR) pathway (*i.e*., through the resection and subsequent invasion and copying of the E_st_ homologous chromosome^46^). According to this model, the proximal break would have also occurred at the limit between the A_p_ and A_d_ sections. Upon inversion of the central fragment of the E_1+2_ chromosome, both breaks would have been repaired and generated inversion E_9_. Repair of the A_p_ section would have, however, taken place through the BIR pathway and using the E_st_ chromosome as template. Repair of the proximal break would have thus resulted in a copy of the A_d_ section of the E_st_ homologue, which would explain the similarity observed between the A_d_ fragments present in the AB, AK and AH2 regions. In contrast, the distal break would have been repaired through the NHEJ pathway. In this case, the presence of the E_1+2_ A_d_ section in the distal break would explain the similarity observed between the A_d_ section present in the AG and GAL regions.

The three models proposed to explain the origin of inversion E_9_ from an E_st_/E_1+2_ heterokaryotype differ in the number and location of double-strand breaks at the inversion breakpoints as well as in the pathway/s used to repair these breaks. Based on the number of breaks, the BIR-NHEJ model would seem the most likely as it only involves two double-strand breaks in a single chromosome whereas the NHEJ-4 model would seem the least likely as it does not only require the highest number of double-strand breaks affecting both homologous chromosomes but also the distal break to have occurred between the same two nucleotides in both homologous chromosomes. Nevertheless, as models also differ in the repair pathways involved, further discrimination among models should await a better characterization of these pathways in the Drosophila genus.

In summary, our study revealed that the molecular genealogy inferred from variation at the A fragment differed from the cytology-based phylogeny of inversions E_1_, E_2_, E_9_ and E_3_ by the clustering of chromosomal arrangements E_[1+2+9]_ and E_1+2+9+3_ with E_st_ instead of with E_1+2_ as expected. To explain this discrepancy, we propose that inversion E_9_ originated in an E_st_/E_1+2_ heterokaryotypic individual, and develop three alternative models for the origin of E_9_ in such a heterokaryotype. This is, to our knowledge, the first documented case where the two homologous chromosomes of a heterokaryotypic individual are required to explain the origin of an inversion. Even though this situation may apply to other inversions, it should be noted that it is the characteristics of the complex here studied —*i.e*., a system with multiple arrangements resulting from the sequential accumulation of overlapping inversions that share a breakpoint at the molecular level— that have permitted its detection.

## Materials and Methods

We used 29 individuals of *D. subobscura* sampled from a wild population at Observatori Fabra (Barcelona, Catalonia, Spain). These individuals had been previously identified as heterokaryotypic for any pair of the five E chromosome arrangements considered in the present work^8^. Their heterokaryotypic status allowed in many cases the independent PCR amplification of the A fragment of each of its two homologous chromosomes (Supplementary Table S1).

Regions spanning the breakpoints were PCR amplified using TaKaRa DNA polymerase (Takara Bio Inc) and newly designed oligonucleotide pairs (Supplementary Table S3). For each amplified region (ranging from 5.4 to 6.6 kb), an ~2-kb long stretch that spans fragment A was sequenced (Fig. 2). Sequence reactions were performed with the ABI PRISM version 3.2 cycle sequencing kit and the sequencing products separated on an ABI PRISM 3730 sequencer. All sequences were obtained on both strands and assembled using the DNASTAR package^47^. Sequences newly obtained have been deposited in the European Nucleotide Archive (ENA) under project number PRJEB33551. The A fragment sequence of *D. guanche* was retrieved from its complete genome sequence^45^ (https://denovo.cnag.cat/genomes/dgua/).

The MUSCLE program in the MEGA7 package^48^ was used for sequence alignment. Genetic differentiation between chromosomal arrangements was measured using the *F*_ST_ statistic^38^ and its statistical significance established using the mstatspop beta version (https://bioinformatics.cragenomica.es/numgenomics/people/sebas/software/software.html) with a total of 10000 random permutations. Summary statistics for nucleotide polymorphism and divergence were obtained using the DnaSP v6 program^49^. MEGA7 was also used to infer the Neighbor-Joining trees using the partial deletion option, in which nucleotide positions with less than 95% site coverage were eliminated before computing the corresponding evolutionary distances using the Jukes and Cantor correction^50^.

## Supplementary information

Supplementary information

## Data Availability

The *D. subobscura* sequences newly obtained have been deposited in the European Nucleotide Archive (ENA) under project number PRJEB33551.
