# Supplementary material for: The molecular genealogy of sequential overlapping inversions implies both homologous chromosomes of a heterokaryotype in an inversion origin
Source: Sci Rep. 2019 Nov 18;9:17009. doi: 10.1038/s41598-019-53582-8 (PMC6861252; doi:10.1038/s41598-019-53582-8)
Supplement: Supplementary file 1 — Supplementary information [file 41598_2019_53582_MOESM1_ESM.pdf]

Supplementary Information

The molecular genealogy of sequential overlapping inversions implies both homologous chromosomes of a heterokaryotype in an inversion origin

Dorcas J. Orengo[1], Eva Puerma[1], U. Cereijo[1, 2], M. Aguadé[1*]

[1]Departament de Genètica, Facultat de Biologia, i Institut de Recerca de la Biodiversitat (IRBio), Universitat de Barcelona, Barcelona, Spain
[2] Present address: Centre for Research in Agricultural Genomics, CSIC-IRTA-UAB-UB, Campus UAB, Bellaterra (Cerdanyola del Vallès) 08193 Barcelona, Spain

*Corresponding author

Supplementary Table S1. Breakpoint regions including fragment A in 29 heterokaryotypic individuals from a wild population. 1

Supplementary Table S2. Nucleotide polymorphisms in the 4243-nt long multiple alignment of fragment A from five different breakpoint regions. 2

Supplementary Table S3. Primer pairs used to amplify fragment A in the different chromosomal arrangements. 10

Supplementary Figure S1. Neighbor-joining tree of the A part sequences of homokaryotypic lines of different E chromosomal arrangements of *Drosophila subobscura*. 11

Supplementary Table S1. Breakpoint regions including fragment A in 29 heterokaryotypic individuals from a wild population.

individual	karyotype		Breakpoint region				
			AB	AG	AK	AH2	GAL
3M	$E_{\underline{1+2}}$	$E_{\underline{1+2+9+12}}$		1	+		1
4M	E_{st}	$E_{\underline{1+2}}$	+	+			
5M	E_{st}	$E_{\underline{1+2+9+12}}$	+		+		+
7M	E_{st}	$E_{\underline{1+2}}$	+	+			
9M	$E_{\underline{1+2}}$	$E_{\underline{1+2+9+3}}$		1		+	1
14M	E_{st}	$E_{\underline{1+2}}$	+	ns			
16M	E_{st}	$E_{\underline{1+2+9+12}}$	+		ns		+
17M	E_{st}	$E_{\underline{1+2+9+3}}$	+			+	ns
18M	$E_{\underline{1+2}}$	$E_{\underline{1+2+9}}$		1	+		1
20M	E_{st}	$E_{\underline{1+2+9+12}}$	ns		+		ns
21M	$E_{\underline{1+2}}$	$E_{\underline{1+2+9+12}}$		1	+		1
23M	E_{st}	$E_{\underline{1+2+9}}$	+		ns		+
24M	E_{st}	$E_{\underline{1+2+9}}$	ns		+		+
25M	E_{st}	$E_{\underline{1+2}}$	+	+			
2H	E_{st}	$E_{\underline{1+2+9+12}}$	+		+		+
11H	E_{st}	$E_{\underline{1+2+9+12}}$	+		ns		ns
16H	E_{st}	$E_{\underline{1+2+9}}$	+		ns		+
17H	E_{st}	$E_{\underline{1+2}}$	+	+			
18H	E_{st}	$E_{\underline{1+2+9+12}}$	+		ns		ns
21H	E_{st}	$E_{\underline{1+2+9+12}}$	+		+		+
23H	$E_{\underline{1+2}}$	$E_{\underline{1+2+9}}$		1	+		1
25H	E_{st}	$E_{\underline{1+2}}$	ns	+			
26H	E_{st}	$E_{\underline{1+2+9+12}}$	+		+		+
27H	E_{st}	$E_{\underline{1+2+9}}$	+		ns		+
30H	E_{st}	$E_{\underline{1+2+9}}$	+		ns		+
31H	$E_{\underline{1+2+9+3}}$	$E_{\underline{1+2+9+12}}$			ns	+	2
50H	$E_{\underline{1+2}}$	$E_{\underline{1+2+9+3}}$		1		+	1
51H	E_{st}	$E_{\underline{1+2}}$	+	+			
52H	$E_{\underline{1+2+9}}$	$E_{\underline{1+2+9+3}}$			+	+	2

+, regions from which fragment A was sequenced
Gray shaded cells indicate regions of a particular individual with two copies of the A fragment neighboring the G part of the corresponding breakpoint region on either the same or different chromosomes. Numbers indicate the number of copies of the AG and/or GAL regions in a particular individual.
ns, not sequenced.

Supplementary Table S2. Nucleotide polymorphisms in the 4243-nt long multiple alignment of fragment A from five different breakpoint regions.

126-391 insertion in AK_52H

~Dpse\snoRNA GA29823 ~Dpse\snoRNA GA29824

Dpse\snoRNA: GA29824

	8	11	14	17	19	26	28	31	32	38	44	45	53	76	392	395	396	398	399	403	404	408	409	413	417	419	420	421	432	442	444	448	451	454	462	468	470	475	488	505	517	518	536	546	549	557	562	568	573	576
AB 4M	A	A	A	A	T	A	A	G	T	G	A	C	A	G	T	C	A	A	A	T	T	C	A	A	C	G	G	T	A	T	C	G	C	A	A	G	A	T	A	T	T	T	C	G	T	C	G	A	G	T
AB 5M	.	.	.	.	.	.	.	.	.	.	.	.	.	.	.	.	.	.	.	.	.	.	.	.	.	.	.	.	.	.	.	.	.	.	.	A	.	.	.	.	.	.	.	.	.	.	.	.	.	.
AB 7M	.	.	.	.	.	.	.	.	.	.	.	.	.	.	.	.	.	.	.	.	.	.	.	.	.	.	.	.	.	.	.	.	.	.	.	.	.	.	.	.	.	.	T	.	.	T	.	.	.	.
AB 14M	.	.	.	.	.	.	.	.	.	.	.	.	.	.	.	T	.	.	.	G	.	T	.	.	.	.	.	.	.	.	.	.	.	.	.	.	.	.	.	.	.	.	.	.	.	.	.	.	.	.
AB 16M	?	?	?	?	?	?	?	?	?	?	?	?	?	?	.	.	.	.	.	.	.	.	.	.	.	.	.	.	.	.	.	.	.	.	.	A	.	.	.	.	.	.	.	.	.	.	.	.	.	.
AB 17M	.	.	.	.	.	.	.	.	.	.	.	.	.	.	.	.	.	.	.	.	.	.	.	.	.	.	.	.	.	.	.	.	.	.	.	.	.	.	.	.	.	.	.	.	.	.	.	.	.	.
AB 23M	.	.	.	.	.	.	.	.	.	.	.	.	.	.	.	.	.	.	.	.	.	.	.	.	.	.	.	.	.	.	.	.	.	.	.	.	.	.	.	.	.	.	T	.	.	.	.	.	.	.
AB 25M	.	.	.	.	.	.	.	.	.	.	.	.	.	.	.	.	.	.	.	G	.	.	.	.	.	.	.	.	.	.	.	.	.	.	.	.	.	.	.	.	A	.	.	.	.	.	.	.	.	.
AB 2H	.	.	.	.	.	.	.	.	.	.	.	.	.	.	.	T	.	.	.	G	.	T	.	.	.	.	.	.	.	.	.	.	.	.	.	.	.	.	.	.	A	.	.	.	.	.	.	.	.	.
AB 11H	.	.	.	.	.	.	.	.	.	.	.	.	.	.	.	.	.	.	.	.	.	.	.	.	.	.	.	.	.	.	.	.	T	.	.	.	.	.	.	.	.	.	.	.	.	.	.	.	.	.
AB 16H	.	.	.	.	.	.	.	.	.	.	.	.	.	.	.	.	.	.	.	G	.	T	.	.	.	.	.	.	.	.	.	.	.	.	.	.	.	.	.	.	A	.	.	.	.	.	.	.	.	.
AB 17H	.	.	.	.	.	.	.	.	.	.	.	.	.	.	.	.	.	.	.	.	.	.	.	.	.	.	.	.	.	.	.	.	.	.	.	.	.	.	.	.	.	.	.	.	.	.	.	.	.	.
AB 18H	.	.	.	.	.	.	.	.	.	.	.	.	.	.	.	.	.	.	.	.	.	.	.	.	.	.	.	.	.	.	.	.	.	.	.	.	.	.	.	.	.	.	.	.	.	.	.	.	.	.
AB 21H	.	.	.	.	A	.	.	.	C	.	.	.	.	.	C	.	.	.	.	A	.	.	.	.	.	.	.	.	.	C	.	.	.	.	T	.	.	.	.	.	A	.	.	.	.	.	.	.	.	.
AB 26H	.	.	.	.	A	.	.	.	C	.	.	.	.	.	C	.	.	.	.	A	.	.	.	.	.	.	.	.	.	C	.	.	.	.	T	.	.	.	.	.	A	.	.	.	.	.	.	.	.	.
AB 27H	.	.	.	.	.	.	.	C	.	.	.	.	.	.	.	.	.	.	.	.	.	.	.	.	.	.	.	.	.	.	.	.	.	.	.	.	.	.	.	.	.	.	.	.	.	.	.	.	.	.
AB 30H	.	.	.	.	.	.	.	.	.	.	.	.	.	.	.	T	.	.	.	G	.	T	.	.	.	.	A	.	.	.	.	.	.	.	.	.	.	.	.	.	.	.	.	.	.	.	.	.	.	.
AB 51H	.	.	.	.	A	.	.	.	C	.	.	.	.	.	C	.	.	.	.	A	.	.	.	.	.	.	.	.	.	C	.	.	.	.	T	.	.	.	.	.	A	.	.	.	.	.	.	.	.	.
AG 4M	T	.	.	.	.	.	G	.	.	.	.	.	.	.	.	T	T	C	G	.	.	.	C	G	T	A	.	C	T	C	A	C	.	C	.	.	C	A	G	.	A	.	.	.	.	.	.	G	A	.
AG 7M	T	.	.	.	.	.	G	.	.	.	.	.	.	.	.	T	T	C	G	.	.	.	C	G	T	A	.	C	T	C	A	C	.	C	.	.	C	A	G	.	A	.	.	.	.	.	.	G	A	.
AG 25M	T	.	.	.	.	.	G	.	.	.	.	.	.	.	.	T	.	C	G	.	.	.	C	G	T	A	.	C	.	C	A	C	.	C	.	.	.	.	G	.	A	A	.	T	.	.	.	G	A	.
AG 17H	T	.	.	.	.	.	G	.	.	.	.	.	.	.	.	T	T	C	G	.	.	.	C	G	T	A	.	C	T	C	A	C	.	C	.	.	C	A	G	.	A	.	.	.	.	.	.	G	A	.
AG 25H	T	.	.	.	.	.	G	.	.	.	.	.	.	.	.	T	T	C	G	.	.	.	C	G	T	A	.	C	T	C	A	C	.	C	.	.	C	A	G	.	A	.	.	.	.	.	.	G	A	.
AG 51H	T	.	.	.	.	.	G	.	.	.	.	.	.	.	.	T	.	C	G	.	.	.	C	G	T	A	.	C	.	C	A	C	.	C	.	.	.	.	G	.	A	A	.	T	.	.	.	G	A	.
GAL 5M	.	.	.	.	.	T	.	.	.	.	.	.	.	.	.	T	.	.	.	.	G	.	.	.	.	.	.	.	.	.	.	.	.	.	.	.	C	.	.	.	A	.	.	.	.	.	C	.	A	.
GAL 16M	.	.	.	.	.	T	.	.	.	.	.	.	.	.	.	T	.	.	.	.	.	.	.	.	.	.	.	.	.	.	.	.	.	.	.	.	C	.	.	.	A	.	.	.	.	.	C	.	A	.
GAL 23M	.	.	.	T	.	T	.	.	.	.	.	.	.	.	.	T	.	.	.	.	G	.	.	.	.	.	.	.	.	.	.	.	.	.	.	.	.	.	.	.	A	.	.	.	.	.	C	.	A	.
GAL 24M	.	.	.	.	.	T	.	A	.	.	.	.	.	.	.	T	.	.	.	.	.	.	.	.	.	.	.	.	.	.	.	.	.	.	.	.	C	.	.	.	A	.	.	.	.	.	C	.	A	.
GAL 2H	.	.	.	.	.	T	.	.	.	.	.	.	.	.	.	T	.	.	.	.	G	.	.	.	.	.	.	.	.	.	.	.	.	.	.	.	.	.	.	.	A	.	.	.	.	.	C	.	A	.
GAL 16H	.	.	.	.	.	T	.	.	.	.	.	.	.	.	.	T	.	.	.	.	.	.	.	.	.	.	.	.	.	C	.	.	.	.	.	.	.	.	.	.	A	.	.	.	.	.	C	.	A	.
GAL 21H	.	.	.	.	.	T	.	.	.	.	.	.	.	.	.	T	.	.	.	.	G	.	.	.	.	.	.	.	.	.	.	.	.	.	.	.	.	.	.	.	A	.	.	.	.	.	C	.	A	.
GAL 26H	.	T	.	.	.	T	.	.	.	A	.	.	T	.	.	T	.	.	.	.	.	.	.	.	.	.	.	.	.	.	.	.	.	.	.	.	C	.	.	.	A	.	.	.	.	.	C	.	A	.
GAL 27H	.	T	.	.	.	T	.	.	.	.	.	.	.	.	.	T	.	.	.	.	G	.	.	.	.	.	.	.	.	.	.	.	.	.	.	.	.	.	.	.	A	.	.	.	.	.	C	.	A	.
GAL 30H	.	.	.	.	.	T	.	.	.	.	.	.	.	.	.	T	.	.	.	.	.	.	.	.	.	.	.	.	.	C	.	.	.	.	.	.	.	.	.	.	A	.	.	.	.	.	C	.	A	.
AK 3M	.	.	.	.	.	.	.	.	.	.	.	.	.	.	.	.	.	.	.	.	.	.	.	.	.	.	.	.	.	.	.	.	.	.	.	.	.	.	.	.	A	.	.	.	.	.	.	.	A	.
AK 5M	.	.	.	.	.	.	.	.	.	.	.	.	.	.	.	.	.	.	.	.	G	.	.	-	T	.	.	.	.	.	.	.	.	.	.	.	.	.	.	.	A	.	.	.	.	.	.	-	A	.
AK 18M	.	.	.	.	.	.	.	.	.	.	.	.	.	.	.	.	.	.	.	.	G	.	.	.	.	.	.	.	.	.	.	.	.	.	.	.	.	.	.	.	A	.	.	.	.	.	.	.	A	.
AK 20M	.	.	.	.	.	.	.	.	.	.	.	.	.	.	.	.	.	.	.	.	.	.	.	.	.	.	.	.	.	.	.	.	.	.	.	.	.	.	.	.	A	.	.	.	.	.	.	.	A	.
AK 21M	.	.	.	.	.	.	.	.	.	.	.	.	.	.	.	.	.	.	.	.	.	.	.	.	.	.	.	.	.	.	.	.	.	.	.	.	.	.	.	.	A	.	.	.	A	.	.	.	A	.
AK 24M	.	.	.	.	.	.	.	.	.	.	.	.	.	.	.	.	.	.	.	.	G	.	.	.	.	.	.	.	.	.	.	.	.	.	.	.	.	.	.	.	A	.	.	.	.	.	.	.	A	.
AK 2H	.	.	.	.	.	.	G	.	.	-	G	T	.	.	.	.	.	.	.	.	G	.	.	.	.	.	.	.	.	.	.	.	.	.	.	.	.	.	.	.	A	.	.	.	.	.	.	.	A	.
AK 21H	.	.	.	.	.	.	.	.	.	.	.	.	.	.	.	.	.	.	.	.	G	.	.	.	.	.	.	.	.	.	.	.	.	.	.	.	.	.	.	.	A	.	.	.	.	.	.	.	A	.
AK 23H	.	.	.	.	.	.	.	.	.	.	.	.	.	.	.	.	.	.	.	.	G	.	.	.	.	.	.	.	.	.	.	.	.	.	.	.	.	.	.	.	A	.	.	.	.	.	.	.	A	.
AK 26H	.	.	.	.	.	.	.	.	.	.	.	.	.	.	.	.	.	.	.	.	G	.	.	-	T	.	.	.	.	.	.	.	.	.	.	.	.	-	.	.	A	.	.	.	.	.	.	.	A	.
AK 52H	.	.	.	.	.	.	.	.	.	.	.	.	.	.	.	.	.	.	.	.	G	.	.	.	.	.	.	.	.	.	.	.	.	.	.	.	.	.	.	.	A	.	.	.	.	.	.	.	A	.
AH2 9M	.	.	.	.	.	.	.	A	.	.	.	.	.	A	.	.	.	.	.	.	.	.	.	.	.	.	.	.	.	.	.	.	.	.	.	.	.	.	.	.	A	.	.	.	.	.	.	.	A	.
AH2 17M	.	.	.	.	.	.	.	A	.	.	.	.	.	A	.	.	.	.	.	.	.	.	.	.	.	.	.	.	.	.	.	.	.	.	.	.	.	.	.	.	A	.	.	.	.	.	.	.	A	.
AH2 31H	.	.	.	.	.	.	.	A	.	.	.	.	.	A	.	.	.	.	.	.	.	.	.	.	.	.	.	.	.	.	.	.	.	.	.	.	.	.	.	A	A	.	.	.	.	.	.	.	A	G
AH2 50H	.	T	C	.	.	T	.	.	.	.	.	.	.	.	.	.	.	.	.	.	.	.	.	.	.	.	.	.	.	.	.	.	.	.	.	.	.	.	.	A	A	.	.	.	.	.	.	.	A	G
AH2 52H	.	.	.	.	.	.	.	A	G	.	.	.	.	A	.	.	.	.	.	.	.	.	.	.	.	.	.	.	.	.	.	.	.	.	.	.	.	.	.	.	A	.	.	.	.	.	.	.	A	.

Horizontal lines separate the A sequences included in the different breakpoint regions (AB, AG, GAL, AK and AH2). A dash indicates a nucleotide deletion and a question mark a non-sequenced site. Grey shadowed sites in a particular group of sequences highlight those sites not analyzed in that group in the complete-deletion option. Boxes group polymorphic sites either affected by the same deletion or not sequenced. Sites with multiple hits are indicated by red numbers when considering all sequences and by red letters when within a particular group of sequences. Horizontal lines above polymorphic sites indicate sites located within homologous sequences of snoRNA genes.

(continued)

Supplementary Table S2. Nucleotide polymorphisms in the 4243-nt long multiple alignment of fragment A from five different breakpoint regions.

685-1459 SGM insertion in AK_20M (between sites 684 and 1471)

Dpse\snoRNA GA29823 (sites 634–673)

	609	634	652	655	669	670	673	674	676	681	684	1471	1493	1500	1502	1503	1509	1510	1511	1527	1529	1533	1537	1538	1540	1541	1542	1543	1545	1547	1548	1549	1551	1553	1556	1560	1571	1577	1581	1582	1586	1589	1592	1596	1597	1605	1609	1612	1613	1614
AB 4M	G	A	A	T	C	G	C	C	C	A	C	A	T	C	C	C	T	C	T	G	A	T	T	A	G	G	G	C	T	C	C	G	C	C	G	T	A	G	G	A	C	A	A	A	A	G	T	T	A	G
AB 5M	.	.	.	.	.	.	.	.	.	.	.	.	.	G	.	.	.	.	.	.	.	.	.	.	.	.	.	.	.	.	.	.	.	.	.	.	.	.	T	.	.	.	.	.	.	A	.	A	.	T
AB 7M	.	.	.	.	.	.	.	.	.	.	.	.	.	G	.	.	.	.	.	.	.	.	.	.	.	.	.	.	.	.	.	.	.	.	.	.	.	.	.	.	.	.	.	.	.	.	.	.	.	.
AB 14M	.	.	.	.	T	.	T	T	-	T	.	.	A	G	.	.	A	.	.	.	G	.	.	G	A	.	C	.	.	.	T	.	.	.	.	.	.	.	.	.	.	.	.	.	.	.	.	.	.	.
AB 16M	.	.	C	.	.	.	.	.	.	.	.	.	.	G	.	.	.	.	.	.	.	.	.	.	.	.	.	.	.	.	.	.	.	.	.	.	.	.	T	.	.	.	.	.	.	A	.	A	.	T
AB 17M	.	.	.	.	.	.	.	.	.	.	.	.	.	G	.	.	.	.	.	.	.	.	.	.	.	.	.	.	.	.	.	.	.	.	.	.	.	.	T	.	.	.	.	.	.	A	.	A	.	T
AB 23M	.	.	.	.	.	.	.	.	.	.	.	.	.	G	.	.	.	.	.	.	.	.	.	.	.	.	.	.	.	.	.	.	.	.	.	.	.	.	.	.	.	.	.	.	.	.	.	.	.	.
AB 25M	.	.	.	.	.	.	.	.	.	.	.	.	.	G	.	.	.	.	.	.	.	.	.	.	.	.	.	.	.	.	.	.	.	.	.	.	.	.	T	.	.	.	.	.	.	A	.	A	.	T
AB 2H	.	.	.	.	T	.	T	T	-	T	.	.	A	G	.	.	A	.	.	.	G	.	.	G	A	.	C	.	.	.	T	.	.	.	.	.	.	.	.	.	.	.	.	.	.	.	.	.	.	.
AB 11H	.	.	.	.	.	.	.	.	.	.	.	.	.	G	.	.	.	.	.	.	.	.	.	.	.	.	.	.	.	.	.	.	.	.	.	.	.	.	T	.	.	.	.	.	.	A	.	A	.	T
AB 16H	.	.	.	.	.	.	.	.	.	.	.	.	.	.	.	.	.	.	.	.	.	.	.	.	.	.	.	T	.	.	.	.	.	.	.	.	.	.	.	.	.	.	.	.	.	.	.	.	.	.
AB 17H	.	.	.	.	.	.	.	.	.	.	.	.	.	G	.	.	.	.	.	.	.	.	.	.	.	.	.	.	.	.	.	.	.	.	.	.	.	.	T	.	.	.	.	.	.	A	.	A	.	T
AB 18H	.	.	.	.	.	.	.	.	.	.	.	.	.	G	.	.	.	.	.	.	.	.	.	.	.	.	.	.	.	.	.	.	.	.	.	.	.	.	T	.	.	.	.	.	.	A	.	A	.	T
AB 21H	.	.	.	.	T	.	T	T	.	T	.	.	.	A	.	A	.	.	.	.	.	.	.	.	.	.	.	.	.	.	.	.	.	A	.	.	.	.	.	.	A	.	.	.	.	.	.	A	.	T
AB 26H	.	.	.	.	T	.	T	T	.	T	.	.	.	A	.	A	.	.	.	.	.	.	.	.	.	.	.	.	.	.	.	.	.	A	.	.	.	.	.	.	A	.	.	.	.	.	.	A	.	T
AB 27H	.	.	.	.	.	.	.	.	.	.	.	.	.	G	.	.	.	.	.	.	.	.	.	.	.	.	.	.	.	.	.	.	.	.	.	.	.	.	.	.	.	.	.	.	.	.	.	.	.	.
AB 30H	.	.	.	.	T	.	T	T	.	T	.	.	.	T	.	A	.	.	.	.	G	.	.	G	A	.	C	.	.	.	T	.	.	.	A	.	.	.	.	.	.	T	.	.	.	.	.	.	.	T
AB 51H	.	.	.	.	T	.	T	T	.	T	.	.	.	A	.	A	.	.	.	.	.	.	.	.	.	.	.	.	.	.	.	.	.	A	.	.	.	.	.	.	A	.	.	.	.	.	.	A	.	T
AG 4M	.	.	.	.	.	.	.	.	.	.	.	.	.	G	.	.	.	.	.	.	.	.	.	.	A	A	.	.	A	.	.	.	.	.	.	.	.	.	.	.	A	.	.	G	.	.	A	A	C	T
AG 7M	.	.	.	.	.	.	.	.	.	.	.	.	.	G	.	.	.	.	.	.	.	.	.	.	A	A	.	.	.	.	.	.	.	.	.	.	.	.	.	.	A	.	.	G	.	.	A	A	C	T
AG 25M	.	.	.	.	.	.	.	.	.	.	.	.	.	G	.	.	.	.	.	.	.	.	.	.	A	A	.	.	.	.	.	.	.	.	.	.	.	.	.	.	A	.	.	G	.	.	A	A	C	T
AG 17H	.	.	.	.	.	.	.	.	.	.	.	.	.	G	.	.	.	.	.	.	.	.	.	.	A	A	.	.	.	.	.	.	.	.	.	.	.	.	.	.	A	.	.	G	.	.	A	A	C	T
AG 25H	.	.	.	.	.	.	.	.	.	.	.	.	.	G	.	.	.	.	.	.	.	.	.	.	A	A	.	.	.	.	.	.	.	.	.	.	.	.	.	.	A	.	.	G	.	.	A	A	C	T
AG 51H	.	.	.	.	.	.	.	.	.	.	.	G	.	G	.	.	.	.	.	.	.	.	.	T	.	.	.	.	.	.	.	.	.	.	.	.	G	.	.	.	A	.	.	G	.	.	A	A	C	T
GAL 5M	.	.	.	.	.	.	.	.	.	.	.	.	.	G	.	.	.	.	.	.	.	.	.	.	.	.	.	.	.	.	.	.	A	.	.	.	.	.	.	.	A	.	.	G	.	.	A	G	C	T
GAL 16M	.	.	.	.	.	.	.	.	G	.	.	.	.	G	.	.	.	-	-	.	.	.	.	.	.	.	.	.	.	A	.	.	.	.	.	.	.	.	.	.	A	.	.	G	.	.	A	A	C	T
GAL 23M	.	.	.	.	.	.	.	.	.	.	.	.	.	G	.	.	.	.	.	.	.	.	.	.	.	.	.	G	.	.	.	.	A	.	.	.	.	.	.	.	A	.	.	G	.	.	A	G	C	T
GAL 24M	.	.	.	.	.	.	.	.	G	.	.	.	.	G	.	.	.	-	-	.	.	.	.	.	.	.	.	.	.	A	.	.	.	.	.	.	.	.	.	.	A	.	.	G	.	.	A	A	C	T
GAL 2H	.	.	.	.	.	.	.	.	.	.	.	.	.	G	.	.	.	T	.	.	.	.	.	.	.	.	.	.	.	.	.	.	.	.	.	.	.	.	A	.	A	.	.	G	G	.	A	G	C	T
GAL 16H	.	.	.	.	.	.	.	.	.	.	.	.	.	G	.	.	.	T	.	.	.	.	.	.	.	.	.	.	.	.	.	.	.	.	.	.	.	.	.	.	A	.	.	G	G	.	A	G	C	T
GAL 21H	.	.	.	.	.	.	.	.	.	.	.	.	.	G	.	.	.	.	.	.	.	.	.	.	.	.	.	.	.	.	.	.	A	.	.	.	.	.	.	.	A	.	.	G	.	.	A	G	C	T
GAL 26H	.	.	.	.	.	.	.	.	.	.	.	.	.	G	.	.	.	T	.	.	.	.	.	.	.	.	.	.	.	.	.	.	.	.	.	.	.	.	.	.	A	.	.	G	.	.	A	G	C	T
GAL 27H	.	.	.	.	.	.	.	.	G	.	.	.	.	G	.	.	.	.	.	.	.	.	.	.	.	.	.	.	.	A	.	.	.	.	.	A	.	.	.	.	A	.	.	G	.	.	A	A	C	T
GAL 30H	.	.	.	.	.	.	.	.	.	.	.	.	.	G	.	.	.	.	.	.	.	.	.	.	.	.	.	.	.	.	.	.	A	.	.	.	.	.	.	.	A	.	.	G	.	.	A	G	C	T
AK 3M	.	.	.	.	.	.	.	.	.	.	.	.	.	G	.	.	.	.	.	.	.	C	C	.	.	.	.	.	.	.	.	.	.	.	.	.	.	.	.	.	.	.	.	.	.	.	.	A	.	T
AK 5M	.	.	.	.	.	A	.	.	.	.	.	.	.	G	.	.	.	.	.	.	.	.	.	.	.	.	.	.	.	.	.	A	.	.	.	.	.	.	.	G	.	.	.	.	.	.	.	A	.	T
AK 18M	.	.	.	.	.	.	.	.	.	.	.	.	.	G	.	.	.	.	.	.	.	C	C	.	.	.	.	.	.	.	.	.	.	.	.	.	.	A	.	.	.	.	T	.	.	.	.	A	.	T
AK 20M	T	G	.	.	.	.	.	.	.	.	.	.	.	G	.	.	.	.	.	.	.	C	.	.	.	.	.	.	.	.	.	.	.	.	.	.	.	A	.	.	.	.	T	.	.	.	.	A	.	.
AK 21M	.	.	.	.	.	.	.	.	.	.	.	.	.	G	.	.	.	.	.	.	.	C	C	.	.	.	.	.	.	.	.	.	.	.	.	.	.	.	.	.	.	.	.	.	.	.	.	A	.	T
AK 24M	.	.	.	.	.	.	.	.	.	.	.	.	.	G	.	.	.	.	.	.	.	C	C	.	.	.	.	.	.	.	.	.	.	.	.	.	.	.	.	G	.	.	.	.	.	.	.	A	.	T
AK 2H	T	G	.	.	.	.	.	.	.	.	.	.	.	G	.	.	.	.	.	.	.	C	.	.	.	.	.	.	.	.	.	.	.	.	.	.	.	A	.	.	.	.	T	.	.	.	.	A	.	.
AK 21H	.	.	.	.	.	.	.	.	A	.	A	.	.	G	.	.	.	.	.	.	.	.	.	.	.	.	.	.	.	.	.	.	.	.	.	.	.	.	.	.	.	.	.	.	.	.	.	A	.	T
AK 23H	.	.	.	.	.	.	.	.	.	.	.	.	.	G	.	.	.	.	.	A	.	.	.	.	.	.	.	.	.	.	.	.	.	.	.	.	.	.	.	.	.	.	.	.	.	.	.	A	.	T
AK 26H	T	G	.	A	.	.	.	.	.	.	.	.	.	G	A	.	.	.	A	.	.	.	.	.	.	.	.	.	.	.	.	A	.	.	.	.	.	A	.	.	.	.	T	.	.	.	.	A	.	T
AK 52H	.	.	.	.	.	.	.	.	.	.	.	.	.	G	A	.	.	.	A	.	.	.	.	.	.	.	.	.	.	.	.	A	.	.	.	.	.	.	.	G	.	.	.	.	.	.	.	A	.	T
AH2 9M	T	G	.	.	.	.	.	.	.	.	.	.	.	G	.	.	.	.	.	.	.	C	.	.	.	.	.	.	.	.	.	.	.	.	.	.	.	.	.	G	.	.	.	.	.	.	.	A	.	T
AH2 17M	T	G	.	.	.	.	.	.	.	.	.	.	.	G	.	.	.	.	.	.	.	C	.	.	.	.	.	.	.	.	.	.	.	.	.	.	.	.	.	G	.	.	.	.	.	.	.	A	.	T
AH2 31H	.	.	.	.	.	A	.	.	.	.	.	.	.	G	.	.	.	.	.	.	.	.	.	.	.	.	.	.	.	.	.	.	.	.	.	.	.	.	.	.	.	.	.	.	.	.	.	A	.	T
AH2 50H	.	.	.	.	.	A	.	.	.	.	.	.	.	G	.	.	.	.	.	.	.	.	.	.	.	.	.	.	.	.	.	.	.	.	.	.	.	.	.	.	.	.	.	.	.	.	.	A	.	T
AH2 52H	T	G	.	.	.	.	.	.	.	.	.	.	.	G	.	.	.	.	.	.	.	C	.	.	.	.	.	.	.	.	.	.	.	.	.	.	.	.	.	G	.	.	.	.	.	.	.	A	.	T

Horizontal lines separate the A sequences included in the different breakpoint regions (AB, AG, GAL, AK and AH2). A dash indicates a nucleotide deletion and a question mark a non-sequenced site. Grey shadowed sites in a particular group of sequences highlight those sites not analyzed in that group in the complete-deletion option. Boxes group polymorphic sites either affected by the same deletion or not sequenced. Sites with multiple hits are indicated by red numbers when considering all sequences and by red letters when within a particular group of sequences. Horizontal lines above polymorphic sites indicate sites located within homologous sequences of snoRNA genes.

Supplementary Table S2. Nucleotide polymorphisms in the 4243-nt long multiple alignment of fragment A from five different breakpoint regions.

								Dpse\snoRNA: GA29822																														Dmel\snoRNA: Me18S-A1061												
	1623	1624	1625	1636	1639	1644	1654	1667	1673	1694	1695	1703	1705	1711	1735	1749	1761	1793	1800	1821	1822	1823	1824	1826	1836	1846	1853	1854	1856	1863	1865	1872	1874	1878	1886	1887	1890	1907	1915	1918	1926	1935	1970	1975	1984	1985	1992	1995	1999	2000
AB 4M	A	C	G	G	G	T	A	A	T	G	G	A	G	T	G	G	G	C	G	A	C	T	T	A	T	G	T	G	A	G	T	T	C	C	G	C	C	T	A	A	C	T	T	C	A	A	C	T	A	C
AB 5M	.	.	.	.	.	.	.	.	.	.	.	.	.	.	.	.	.	.	.	.	.	.	.	.	.	.	.	.	.	.	.	.	.	.	.	.	.	.	G	.	.	.	.	.	.	.	.	.	.	.
AB 7M	.	.	.	A	.	.	.	.	.	.	.	.	.	.	.	.	.	.	.	T	.	.	.	.	.	.	A	.	C	.	.	.	.	.	.	.	.	.	G	.	.	.	.	.	.	.	.	.	.	.
AB 14M	.	.	.	.	.	.	.	.	.	.	.	.	.	.	.	.	.	.	.	.	.	.	.	.	.	.	.	.	.	.	.	.	.	.	.	.	.	.	.	.	.	.	.	.	.	.	.	.	.	.
AB 16M	.	.	.	.	.	.	.	.	.	.	.	.	.	.	.	.	.	.	.	.	.	.	.	.	.	.	.	.	.	.	.	.	.	.	.	.	.	.	G	.	.	.	.	.	.	.	.	.	.	.
AB 17M	.	.	.	.	.	.	.	.	.	.	.	.	.	.	.	.	.	.	.	.	.	.	.	.	.	.	.	.	.	.	.	.	.	.	.	.	.	.	G	.	.	.	.	.	.	.	.	.	.	.
AB 23M	.	.	.	A	.	.	.	.	.	.	.	.	.	.	.	.	.	.	.	T	.	.	.	.	.	.	A	.	C	.	.	.	.	.	.	.	.	.	G	.	.	.	.	.	.	.	.	.	.	.
AB 25M	.	.	.	.	.	.	.	.	.	.	.	.	.	.	.	.	.	.	.	.	.	.	.	.	.	.	.	.	.	.	.	.	.	.	.	.	.	.	G	.	.	.	.	.	.	.	.	.	.	.
AB 2H	.	.	.	.	.	.	.	.	.	.	.	.	.	.	.	.	.	.	.	.	.	.	.	.	.	.	.	.	.	.	.	.	.	.	.	.	A	.	G	.	.	.	.	.	.	.	.	.	.	.
AB 11H	.	.	.	.	.	.	.	.	.	.	.	.	.	.	.	.	.	.	.	.	.	.	.	.	.	.	.	.	.	.	.	.	.	.	.	.	.	.	G	.	.	.	.	.	.	.	.	.	.	.
AB 16H	.	.	.	.	.	.	.	.	.	.	.	.	.	.	.	.	T	T	.	.	.	.	.	.	.	.	.	.	.	.	.	.	.	.	.	.	.	.	G	.	.	.	.	.	.	.	.	.	.	.
AB 17H	.	.	.	.	.	.	.	.	.	.	.	.	.	.	.	.	.	.	.	.	.	.	.	.	.	.	.	.	.	.	.	.	.	.	.	.	.	.	G	.	.	.	.	.	.	.	.	.	.	.
AB 18H	.	.	.	.	.	.	.	.	.	.	.	.	.	.	.	.	.	.	.	.	.	.	.	.	.	.	.	.	.	.	.	.	.	.	.	.	.	.	G	.	.	.	.	.	.	.	.	.	.	.
AB 21H	.	.	.	.	.	.	.	.	.	.	.	.	.	.	.	.	.	.	.	.	.	.	.	.	.	.	.	.	.	.	.	.	.	.	.	.	.	.	G	.	.	.	.	.	.	.	.	.	.	.
AB 26H	.	.	.	.	.	.	.	.	.	.	.	.	.	.	.	.	.	.	.	.	.	.	.	.	.	.	.	.	.	.	.	.	.	.	.	.	.	.	G	.	.	.	.	.	.	.	.	.	.	.
AB 27H	.	.	.	.	.	.	.	.	.	.	.	.	.	.	.	.	.	.	.	.	.	.	.	.	.	.	.	.	.	.	.	.	.	.	.	.	.	.	.	.	.	.	.	.	.	.	.	.	.	.
AB 30H	.	.	.	.	.	.	.	.	.	.	.	.	.	.	.	.	.	.	.	.	.	.	.	.	.	.	.	.	.	.	.	.	.	.	.	.	.	.	G	.	.	.	.	.	.	.	.	.	.	.
AB 51H	.	.	.	.	.	.	.	.	.	.	.	.	.	.	.	.	.	.	.	.	.	.	.	.	.	.	.	.	.	.	.	.	.	.	.	.	.	.	G	.	.	.	.	.	.	.	.	.	.	.
AG 4M	.	A	.	.	.	C	.	.	.	.	.	.	.	.	.	.	A	.	.	.	.	A	G	G	.	A	.	T	C	C	.	A	A	.	C	T	.	C	G	.	.	.	.	.	.	T	T	C	T	G
AG 7M	.	A	.	.	.	C	.	.	.	.	.	.	.	.	.	.	A	.	.	.	.	A	G	G	.	A	.	T	C	C	.	A	A	.	C	T	.	C	G	.	.	.	.	.	.	T	T	C	T	G
AG 25M	.	A	.	.	.	C	.	.	.	.	.	.	.	.	.	.	A	.	.	.	.	A	G	G	.	A	.	T	C	C	.	A	A	.	C	T	.	C	G	.	.	.	.	.	.	T	T	C	T	G
AG 17H	.	A	.	.	.	C	.	.	.	.	.	.	.	.	.	.	A	.	.	.	.	A	G	G	.	A	.	T	C	C	.	A	A	.	C	T	.	C	G	.	.	.	.	.	.	T	T	C	T	G
AG 25H	.	A	.	.	.	C	.	.	.	.	.	.	.	.	.	.	A	.	.	.	.	A	G	G	.	A	.	T	C	C	.	A	A	.	C	T	.	C	G	.	.	.	.	.	.	T	T	C	T	G
AG 51H	.	A	.	.	.	C	.	.	.	.	.	.	.	.	.	.	A	.	.	.	A	A	A	G	.	A	.	T	C	C	.	.	A	.	C	T	T	C	G	.	.	.	.	.	.	T	T	C	.	.
GAL 5M	.	A	.	.	.	C	.	.	.	.	.	.	.	.	.	.	A	.	.	.	.	A	G	G	.	A	.	T	C	C	.	A	A	.	C	T	.	C	G	.	.	.	.	.	.	T	T	C	T	G
GAL 16M	.	A	.	.	.	C	C	.	.	T	T	.	.	.	.	.	A	.	.	.	.	A	G	G	.	A	.	T	C	C	.	A	A	.	C	T	.	.	G	.	.	.	.	.	.	T	T	C	T	G
GAL 23M	.	A	.	.	.	C	.	.	.	.	.	.	.	.	.	.	A	.	.	.	.	A	G	G	.	A	.	T	C	C	.	A	A	.	C	T	.	C	G	.	.	.	.	.	.	T	T	C	T	G
GAL 24M	.	A	.	.	.	C	C	.	.	T	T	.	.	.	.	.	A	.	.	.	.	A	G	G	.	A	.	T	C	C	.	A	A	.	C	T	.	.	G	.	.	.	.	.	.	T	T	C	T	G
GAL 2H	T	G	.	.	.	C	.	.	.	.	.	.	.	.	.	.	A	.	.	.	A	A	A	G	.	A	.	T	C	C	.	A	A	.	C	T	.	C	G	.	.	.	.	.	.	T	T	C	T	G
GAL 16H	T	G	.	.	.	C	.	.	.	.	.	.	.	.	A	.	A	.	.	.	A	A	A	G	.	A	.	T	C	C	.	A	A	.	C	T	.	.	G	.	.	.	.	.	.	T	T	C	T	G
GAL 21H	.	A	.	.	.	C	.	.	.	.	.	.	.	.	.	.	A	.	.	.	.	A	G	G	.	A	.	T	C	C	.	A	A	.	C	T	.	C	G	.	.	.	.	.	.	T	T	C	T	G
GAL 26H	.	A	.	.	.	C	.	.	.	.	.	.	.	.	.	.	A	.	.	.	.	A	G	G	.	A	.	T	C	C	.	A	A	.	C	T	.	C	G	.	.	.	.	.	.	T	T	C	T	G
GAL 27H	.	A	.	.	.	C	C	.	.	.	.	.	.	.	A	.	A	.	.	.	A	A	A	G	.	A	.	T	C	C	.	A	A	.	C	T	.	C	G	.	.	.	.	.	.	T	T	C	T	G
GAL 30H	.	A	.	.	.	C	.	.	.	.	.	.	.	.	.	.	A	.	.	.	.	A	G	G	.	A	.	T	C	C	.	A	A	.	C	T	.	C	G	.	.	.	.	.	.	T	T	C	T	G
AK 3M	.	.	.	.	.	.	.	.	.	.	.	T	.	A	.	.	.	.	A	.	.	.	.	.	.	.	.	.	.	.	.	.	.	.	.	.	.	.	G	C	.	.	C	.	.	.	.	.	.	.
AK 5M	.	.	.	.	.	.	.	.	.	.	.	.	.	.	.	.	.	.	.	.	.	.	.	.	.	.	.	.	.	.	.	.	.	.	.	.	.	.	G	C	T	.	C	.	.	.	.	.	.	.
AK 18M	.	.	.	.	.	.	.	.	.	.	.	.	.	.	.	.	.	.	A	.	.	.	.	.	.	.	.	.	.	.	.	.	.	.	.	.	.	.	G	C	.	.	C	.	.	.	.	.	.	.
AK 20M	.	.	.	.	.	.	.	.	G	.	.	.	.	.	.	C	.	.	.	.	.	.	.	.	.	.	.	T	.	.	A	.	.	.	.	.	.	.	G	C	.	.	C	.	.	.	.	.	.	.
AK 21M	.	.	.	.	.	.	.	.	.	.	.	T	.	A	.	.	.	.	A	.	.	.	.	.	.	.	.	.	.	.	.	.	.	.	.	.	.	.	G	C	.	.	C	.	.	.	.	.	.	.
AK 24M	.	.	.	.	.	.	.	.	.	.	.	.	.	A	.	.	.	.	A	.	.	.	.	.	.	.	.	.	.	.	.	.	.	.	.	.	.	.	G	C	.	.	C	.	.	.	.	.	.	.
AK 2H	.	.	.	.	.	.	.	.	G	.	.	.	.	.	.	C	.	.	.	.	.	.	.	.	.	.	.	.	G	.	.	.	.	.	.	.	.	.	G	C	.	.	C	.	.	.	.	.	.	.
AK 21H	.	.	.	.	C	.	.	.	.	.	.	.	.	.	.	C	.	.	A	.	.	.	.	.	A	.	.	.	G	.	.	.	.	A	.	.	.	.	G	C	.	.	C	T	C	.	.	.	.	.
AK 23H	.	.	.	.	.	.	.	.	.	.	.	.	.	A	.	.	.	.	A	.	.	.	.	.	.	.	.	.	.	.	.	.	.	.	.	.	.	.	G	C	.	.	-	-	-	-	.	.	.	.
AK 26H	.	.	C	.	.	.	.	.	G	.	.	.	.	.	.	.	.	.	.	.	.	.	.	.	.	.	.	.	.	.	.	.	.	.	.	.	.	.	G	C	T	.	-	-	-	-	.	.	.	.
AK 52H	.	.	.	.	.	.	.	T	.	.	.	.	.	A	.	.	.	.	.	.	.	.	.	.	.	.	.	.	.	-	-	-	-	-	.	.	.	.	G	C	.	.	C	.	C	.	.	.	.	.
AH2 9M	.	.	.	.	.	.	.	.	.	.	.	.	.	.	.	.	.	.	A	.	.	.	.	.	.	.	.	.	.	.	.	.	.	.	.	.	.	.	G	C	.	.	.	.	.	.	.	.	.	.
AH2 17M	.	.	.	.	.	.	.	.	.	.	.	.	.	.	.	.	.	.	A	.	.	.	.	.	.	.	.	.	.	.	.	.	.	.	.	.	.	.	G	C	.	.	.	.	.	.	.	.	.	.
AH2 31H	.	.	.	.	.	.	.	.	.	.	.	.	A	.	.	.	.	.	A	.	.	.	.	.	.	.	.	.	.	.	.	.	.	.	.	.	.	.	G	C	.	C	.	.	.	.	.	.	.	.
AH2 50H	.	.	.	.	.	.	.	.	.	.	.	.	A	.	.	.	.	.	A	.	.	.	.	.	.	.	.	.	.	.	.	.	.	.	.	.	.	.	G	C	.	C	.	.	.	.	.	.	.	.
AH2 52H	.	.	.	.	.	.	.	.	.	.	.	.	.	.	.	.	.	.	A	.	.	.	.	.	.	.	.	.	.	.	.	.	.	.	.	.	.	.	G	C	.	.	.	.	.	.	.	.	.	.

Horizontal lines separate the A sequences included in the different breakpoint regions (AB, AG, GAL, AK and AH2). A dash indicates a nucleotide deletion and a question mark a non-sequenced site. Grey shadowed sites in a particular group of sequences highlight those sites not analyzed in that group in the complete-deletion option. Boxes group polymorphic sites either affected by the same deletion or not sequenced. Sites with multiple hits are indicated by red numbers when considering all sequences and by red letters when within a particular group of sequences. Horizontal lines above polymorphic sites indicate sites located within homologous sequences of snoRNA genes.

Supplementary Table S2. Nucleotide polymorphisms in the 4243-nt long multiple alignment of fragment A from five different breakpoint regions.

2051-2274 Inserttion in AG (and GAL)

	2001	2005	2006	2007	2009	2018	2020	2021	2022	2023	2026	2051	2066	2068	2107	2128	2169	2186	2211	2231	2251	2252	2254	2265	2279	2280	2283	2292	2293	2296	2299	2309	2317	2320	2321	2327	2335	2356	2363	2370	2372	2373	2374	2375	2378	2384	2385	2391	2393	2395
AB 4M	A	T	A	A	G	T	C	A	T	A	T	-	-	-	-	-	-	-	-	-	-	-	-	-	G	G	G	A	T	A	A	-	-	-	-	A	T	T	T	A	C	A	G	T	A	T	G	C	T	T
AB 5M	.	.	.	.	.	.	.	.	.	.	.	-	-	-	-	-	-	-	-	-	-	-	-	-	.	.	.	.	.	.	.	-	-	-	-	.	.	.	A	.	.	.	.	.	.	.	.	.	.	G
AB 7M	.	.	.	.	.	.	.	.	.	.	.	-	-	-	-	-	-	-	-	-	-	-	-	-	.	.	.	.	.	.	.	T	C	A	G	.	.	.	.	.	.	.	.	.	.	.	.	.	.	G
AB 14M	.	.	.	.	.	.	.	.	.	.	.	-	-	-	-	-	-	-	-	-	-	-	-	-	.	.	.	.	.	.	.	-	-	-	-	.	.	.	.	.	.	.	.	.	.	.	.	.	.	.
AB 16M	.	.	.	.	.	.	.	.	.	.	.	-	-	-	-	-	-	-	-	-	-	-	-	-	.	.	.	.	.	.	.	-	-	-	-	.	.	.	A	.	.	.	.	.	.	.	.	.	.	G
AB 17M	.	.	.	.	.	.	.	.	.	.	.	-	-	-	-	-	-	-	-	-	-	-	-	-	.	.	.	.	.	.	.	-	-	-	-	.	.	.	A	.	.	.	.	.	.	.	.	.	.	G
AB 23M	.	.	.	.	.	.	.	.	.	.	.	-	-	-	-	-	-	-	-	-	-	-	-	-	.	.	.	.	.	.	.	T	C	A	G	.	.	.	.	.	.	.	.	.	.	.	.	.	.	G
AB 25M	.	.	.	.	.	.	.	.	.	.	.	-	-	-	-	-	-	-	-	-	-	-	-	-	.	.	.	.	.	.	.	-	-	-	-	.	.	.	A	.	.	.	.	.	.	.	.	.	.	G
AB 2H	.	.	.	.	.	.	.	.	.	.	.	-	-	-	-	-	-	-	-	-	-	-	-	-	.	.	.	.	.	.	.	-	-	-	-	.	.	.	A	.	.	.	.	.	.	.	.	.	.	G
AB 11H	.	.	.	.	.	.	.	.	.	.	.	-	-	-	-	-	-	-	-	-	-	-	-	-	.	.	.	.	.	.	.	-	-	-	-	.	.	.	A	.	.	.	.	.	.	.	.	.	.	G
AB 16H	.	.	.	.	.	.	A	.	.	.	.	-	-	-	-	-	-	-	-	-	-	-	-	-	.	.	.	.	.	.	.	-	-	-	-	.	.	.	A	.	.	.	.	.	.	.	.	.	.	G
AB 17H	.	.	.	.	.	.	.	.	.	.	.	-	-	-	-	-	-	-	-	-	-	-	-	-	.	.	.	.	.	.	.	-	-	-	-	.	.	.	.	.	.	.	.	.	.	.	.	.	.	.
AB 18H	.	.	.	.	.	.	.	.	.	.	.	-	-	-	-	-	-	-	-	-	-	-	-	-	.	.	.	.	.	.	.	-	-	-	-	.	.	.	A	.	.	.	.	.	.	.	.	.	.	G
AB 21H	.	.	.	.	.	.	.	.	.	.	.	-	-	-	-	-	-	-	-	-	-	-	-	-	.	.	.	.	.	.	.	-	-	-	-	.	.	.	A	.	.	.	.	.	.	.	.	.	.	G
AB 26H	.	.	.	.	.	.	.	.	.	.	.	-	-	-	-	-	-	-	-	-	-	-	-	-	.	.	A	.	.	.	.	-	-	-	-	.	.	.	A	.	.	.	.	.	.	.	.	.	.	G
AB 27H	.	-	-	-	.	.	.	.	.	.	.	-	-	-	-	-	-	-	-	-	-	-	-	-	.	.	.	.	.	.	.	-	-	-	-	.	.	.	.	.	.	.	.	.	.	.	.	.	.	.
AB 30H	.	.	.	.	.	.	.	.	.	.	.	-	-	-	-	-	-	-	-	-	-	-	-	-	.	.	.	.	.	.	.	-	-	-	-	.	.	.	A	.	.	.	.	.	.	.	.	.	.	G
AB 51H	.	.	.	.	.	.	.	.	.	.	.	-	-	-	-	-	-	-	-	-	-	-	-	-	.	.	A	.	.	.	.	-	-	-	-	.	.	.	A	.	.	.	.	.	.	.	.	.	.	G
AG 4M	T	.	G	.	.	-	-	-	.	G	.	A	C	G	C	C	G	G	C	G	C	A	C	C	T	A	.	.	C	.	C	G	C	G	G	.	.	.	A	.	T	.	.	.	.	.	.	.	.	G
AG 7M	T	.	G	.	.	-	-	-	.	G	.	A	C	G	C	C	G	G	C	G	C	A	C	C	T	A	.	.	C	.	C	G	C	G	G	.	.	.	A	.	T	.	.	.	T	.	.	.	.	G
AG 25M	T	.	G	.	.	-	-	-	.	G	.	A	C	G	C	C	G	G	C	G	C	A	C	C	T	A	.	.	C	.	C	G	C	G	G	.	.	.	A	.	T	.	.	.	.	.	.	.	.	G
AG 17H	T	.	G	.	.	-	-	-	.	G	.	A	C	G	C	C	G	G	C	G	C	A	C	C	T	A	.	.	C	.	C	G	C	G	G	.	.	.	A	.	T	.	.	.	.	.	.	.	.	G
AG 25H	T	.	G	.	.	-	-	-	.	G	.	A	C	G	C	C	G	G	C	G	C	A	C	C	T	A	.	.	C	.	C	G	C	G	G	.	.	.	A	.	T	.	.	.	.	.	.	.	.	G
AG 51H	.	.	G	T	T	G	.	T	.	.	.	-	-	-	-	-	-	-	-	G	G	G	C	C	A	A	.	.	C	.	C	G	C	A	G	T	.	.	A	.	T	.	.	.	.	.	.	.	.	G
GAL 5M	T	.	G	.	.	-	-	-	.	.	.	-	T	T	T	C	A	G	T	T	C	A	C	C	A	A	.	.	C	.	C	G	T	A	A	.	.	.	A	.	T	.	.	.	.	.	.	.	.	G
GAL 16M	T	.	G	.	.	-	-	-	.	.	.	T	T	T	T	C	A	G	C	T	C	A	C	C	A	A	.	.	C	.	C	G	T	A	A	.	.	.	A	.	T	.	.	.	.	.	.	.	A	G
GAL 23M	T	.	G	.	.	-	-	-	.	.	.	A	C	G	C	C	A	G	C	T	C	A	C	C	A	A	.	.	C	.	C	G	T	A	A	.	.	.	A	.	T	.	.	.	.	.	.	.	.	G
GAL 24M	T	.	G	.	.	-	-	-	.	.	.	T	T	T	T	C	A	G	C	T	C	A	C	A	A	A	.	.	C	.	C	G	T	A	A	.	.	.	A	.	T	.	.	.	.	.	.	.	A	G
GAL 2H	T	.	G	.	.	-	-	-	.	.	.	-	T	T	C	C	A	G	C	T	C	A	C	C	A	A	.	.	C	.	C	G	T	A	A	.	.	.	A	.	T	.	.	.	.	.	.	.	.	G
GAL 16H	T	.	G	.	.	-	-	-	.	.	.	C	T	T	C	A	A	T	C	T	C	A	C	C	A	A	.	.	C	.	C	G	T	A	A	.	.	.	A	.	T	.	.	.	.	.	.	.	A	G
GAL 21H	T	.	G	.	.	-	-	-	.	.	.	C	T	T	C	C	A	G	C	G	C	A	C	C	A	A	.	.	C	.	C	G	T	A	A	.	.	.	A	.	T	.	.	.	.	.	.	.	.	G
GAL 26H	T	.	G	.	.	-	-	-	.	.	.	-	T	T	T	C	A	G	C	T	C	A	C	C	A	A	.	.	C	.	C	G	T	A	A	.	.	.	A	.	T	.	.	.	.	.	.	.	.	G
GAL 27H	T	.	G	.	.	-	-	-	C	.	.	-	T	T	C	C	A	G	C	G	C	A	T	C	A	A	.	.	C	.	C	G	T	A	A	.	.	A	A	.	T	.	.	.	.	.	.	.	A	G
GAL 30H	T	.	G	.	.	-	-	-	.	.	.	C	T	T	C	C	A	G	C	T	C	A	C	C	A	A	.	.	C	.	C	G	T	A	A	.	.	.	A	.	T	.	.	.	.	.	.	.	A	G
AK 3M	.	.	.	.	.	.	.	.	.	.	.	-	-	-	-	-	-	-	-	-	-	-	-	-	.	.	.	.	.	T	.	G	C	A	G	.	.	.	A	.	T	.	T	-	-	-	-	-	-	-
AK 5M	.	.	.	.	.	.	.	.	.	.	.	-	-	-	-	-	-	-	-	-	-	-	-	-	.	A	.	G	.	.	.	G	C	A	G	.	.	.	A	.	T	.	.	.	.	.	.	.	.	G
AK 18M	.	.	.	.	.	.	.	.	.	.	A	-	-	-	-	-	-	-	-	-	-	-	-	-	.	.	.	.	.	T	.	G	C	A	G	.	.	.	A	.	T	.	T	-	-	-	-	-	-	-
AK 20M	.	.	.	.	.	.	.	.	.	.	.	-	-	-	-	-	-	-	-	-	-	-	-	-	.	.	.	.	.	.	.	G	C	A	G	.	.	.	A	T	T	.	.	.	.	.	.	.	.	G
AK 21M	.	.	.	.	.	.	.	.	.	.	.	-	-	-	-	-	-	-	-	-	-	-	-	-	.	.	.	.	.	T	.	G	C	A	G	.	.	.	A	.	T	.	T	-	-	-	-	-	-	-
AK 24M	.	.	.	.	.	.	.	.	.	.	.	-	-	-	-	-	-	-	-	-	-	-	-	-	.	.	.	.	.	T	.	G	C	A	G	.	.	.	A	.	T	.	T	-	-	-	-	-	-	-
AK 2H	.	.	.	.	.	.	.	.	.	.	.	-	-	-	-	-	-	-	-	-	-	-	-	-	.	.	.	.	.	.	.	G	C	A	G	.	.	.	A	.	T	.	.	.	.	C	T	.	.	G
AK 21H	.	.	.	.	.	.	.	.	.	.	.	-	-	-	-	-	-	-	-	-	-	-	-	-	.	.	.	.	.	.	.	G	C	A	G	.	.	.	A	.	T	.	.	G	.	.	.	T	.	G
AK 23H	.	.	.	.	.	.	.	.	.	.	.	-	-	-	-	-	-	-	-	-	-	-	-	-	.	.	.	.	.	T	.	G	C	A	G	.	.	.	A	.	T	.	T	-	-	-	-	-	-	-
AK 26H	.	.	.	.	.	.	.	.	.	.	.	-	-	-	-	-	-	-	-	-	-	-	-	-	.	.	.	.	.	.	.	G	C	A	G	.	.	.	A	.	T	.	.	G	.	.	.	.	.	G
AK 52H	.	.	.	.	.	.	.	.	.	.	.	-	-	-	-	-	-	-	-	-	-	-	-	-	.	.	.	.	.	.	.	G	C	A	G	.	.	.	A	.	T	.	.	.	.	.	.	.	.	G
AH2 9M	.	.	.	.	.	.	.	.	.	.	.	-	-	-	-	-	-	-	-	-	-	-	-	-	.	.	.	.	.	.	.	G	C	A	G	.	G	.	A	.	T	.	.	G	.	.	.	.	.	G
AH2 17M	.	.	.	.	.	.	.	.	.	.	.	-	-	-	-	-	-	-	-	-	-	-	-	-	.	.	.	.	.	.	.	G	C	A	G	.	G	.	A	.	T	.	.	G	.	.	.	.	.	G
AH2 31H	.	C	.	.	.	.	.	.	.	.	.	-	-	-	-	-	-	-	-	-	-	-	-	-	.	.	.	.	.	.	.	G	C	A	G	.	.	.	A	.	T	G	.	G	.	.	.	.	.	G
AH2 50H	.	C	.	.	.	.	.	.	.	.	.	-	-	-	-	-	-	-	-	-	-	-	-	-	.	.	.	.	.	.	.	G	C	A	G	.	.	.	A	.	T	G	.	G	.	.	.	.	.	G
AH2 52H	.	.	.	.	.	.	.	.	.	.	.	-	-	-	-	-	-	-	-	-	-	-	-	-	.	.	.	.	.	.	.	G	C	A	G	.	G	.	A	.	T	.	.	G	.	.	.	.	.	G

Horizontal lines separate the A sequences included in the different breakpoint regions (AB, AG, GAL, AK and AH2). A dash indicates a nucleotide deletion and a question mark a non-sequenced site. Grey shadowed sites in a particular group of sequences highlight those sites not analyzed in that group in the complete-deletion option. Boxes group polymorphic sites either affected by the same deletion or not sequenced. Sites with multiple hits are indicated by red numbers when considering all sequences and by red letters when within a particular group of sequences. Horizontal lines above polymorphic sites indicate sites located within homologous sequences of snoRNA genes.

Supplementary Table S2. Nucleotide polymorphisms in the 4243-nt long multiple alignment of fragment A from five different breakpoint regions.

2612-3419 SGM insertion in some AK (between sites 2607 and 3058)

Dana\snoRNA: GF26125 (over sites 2427–2490); Dana\snoRNA: GF26126 (over sites 3488–3566)

	2403	2405	2406	2410	2413	2423	2427	2431	2480	2487	2489	2490	2500	2516	2517	2525	2527	2528	2538	2552	2563	2575	2582	2595	2603	2607	3058	3176	3295	3393	3424	3427	3431	3436	3446	3465	3469	3487	3488	3542	3556	3563	3566	3571	3574	3577	3578	3580	3582	3592
AB 4M	T	A	C	A	T	A	G	C	C	C	T	A	G	G	G	A	T	T	T	C	C	A	C	G	T	A	-	-	-	-	T	T	C	A	T	C	G	C	A	A	G	T	A	T	T	A	T	A	T	A
AB 5M	.	.	.	.	A	.	.	.	.	.	.	T	.	.	.	C	.	G	.	.	.	G	.	.	A	C	-	-	-	-	A	.	.	.	.	.	.	.	.	.	.	.	.	.	.	.	.	.	.	.
AB 7M	.	.	.	.	.	.	.	.	.	.	.	T	.	.	.	C	G	G	.	.	.	G	.	A	A	.	-	-	-	-	A	.	.	.	.	.	.	.	.	.	.	.	.	.	.	.	.	.	.	.
AB 14M	.	.	.	.	.	.	.	.	.	.	.	.	.	.	.	.	.	.	.	.	.	G	.	.	.	.	-	-	-	-	.	.	.	.	.	.	.	.	.	.	.	.	.	.	.	.	.	.	.	.
AB 16M	.	.	.	.	A	.	.	.	.	.	.	T	.	.	.	C	.	G	A	.	.	G	.	.	A	.	-	-	-	-	A	.	.	.	.	.	.	.	.	.	.	.	.	.	.	.	.	.	.	.
AB 17M	.	.	.	.	A	.	.	.	.	.	.	T	.	.	.	C	.	G	.	.	.	G	.	.	A	.	-	-	-	-	A	.	.	.	.	A	.	.	.	.	.	.	.	.	.	.	.	.	.	.
AB 23M	.	.	.	.	.	.	.	.	.	.	.	T	.	.	.	C	G	G	.	.	.	G	.	A	A	.	-	-	-	-	A	.	A	.	.	.	.	.	.	.	.	.	.	.	.	.	.	.	.	.
AB 25M	.	.	.	.	A	.	.	.	.	.	.	T	.	.	.	C	.	G	.	.	.	G	.	.	A	.	-	-	-	-	A	.	.	.	.	A	.	.	.	.	.	.	.	.	.	.	.	.	.	.
AB 2H	.	.	.	.	A	.	.	.	.	.	.	.	.	.	.	.	.	.	.	.	.	G	.	.	.	.	-	-	-	-	.	.	.	.	.	.	.	.	.	.	.	.	.	.	.	.	.	.	.	.
AB 11H	.	.	.	.	A	.	.	.	.	.	.	T	.	.	.	C	.	G	.	.	.	G	.	.	A	.	-	-	-	-	A	.	.	.	.	A	.	.	.	.	.	.	.	.	.	.	.	.	.	.
AB 16H	.	.	.	.	.	.	.	.	.	.	.	.	.	.	.	C	.	G	.	.	.	G	.	.	A	C	-	-	-	-	A	.	.	.	.	.	.	.	.	.	.	.	.	.	.	.	.	.	.	.
AB 17H	.	.	.	.	.	.	.	.	.	.	.	T	.	A	.	C	.	G	.	.	.	G	.	.	A	.	-	-	-	-	A	.	.	.	.	.	.	.	.	.	.	.	.	.	.	.	.	.	.	.
AB 18H	.	.	.	.	A	.	.	.	.	.	.	T	.	.	.	C	.	G	.	.	.	G	.	.	A	C	-	-	-	-	A	.	.	.	.	.	.	.	.	.	.	.	.	.	.	.	.	.	.	.
AB 21H	.	.	.	.	A	.	.	A	.	.	.	.	.	.	.	.	.	.	.	.	.	.	.	.	.	.	-	-	-	-	.	.	.	.	.	.	.	.	.	.	.	.	.	.	.	.	.	.	.	.
AB 26H	.	.	.	.	A	.	.	.	.	.	.	.	.	.	.	.	.	.	.	.	.	G	.	.	.	.	-	-	-	-	.	.	.	.	.	.	.	.	.	.	.	.	.	.	.	.	.	.	.	.
AB 27H	.	.	.	.	.	.	.	.	.	.	.	T	.	A	.	C	.	G	A	.	.	G	.	.	A	.	-	-	-	-	A	.	.	.	.	.	.	.	.	.	.	.	.	.	.	.	.	.	.	.
AB 30H	.	.	.	.	A	.	.	.	.	.	.	.	.	.	.	.	.	.	.	.	.	.	.	.	.	.	-	-	-	-	.	.	.	.	C	.	.	.	.	.	.	.	.	.	.	.	.	.	.	.
AB 51H	.	.	.	.	A	.	.	.	.	.	.	.	.	.	.	.	.	.	.	.	.	G	.	.	.	.	-	-	-	-	.	.	.	.	.	.	.	.	.	.	.	.	.	.	.	.	.	.	.	.
AG 4M	.	.	.	.	.	.	.	.	.	.	.	T	T	.	.	C	.	G	.	A	.	G	.	.	A	.	-	-	-	-	A	C	.	.	.	.	.	.	.	.	.	.	.	.	.	.	.	.	.	C
AG 7M	.	.	.	.	.	.	.	.	.	.	.	T	T	.	.	C	.	G	.	A	.	G	.	.	A	.	-	-	-	-	A	C	.	.	.	.	.	.	.	.	.	.	.	.	.	.	.	.	.	C
AG 25M	.	.	.	.	.	.	.	.	.	.	.	T	T	.	.	C	.	G	.	A	.	G	.	.	A	.	-	-	-	-	A	C	.	.	.	.	.	.	.	.	.	.	.	.	.	.	.	.	.	C
AG 17H	.	.	.	.	.	.	.	.	.	.	.	T	T	.	.	C	.	G	.	A	.	G	.	.	A	.	-	-	-	-	A	C	.	.	.	.	.	.	.	.	.	.	.	.	.	.	.	.	.	C
AG 25H	.	.	.	.	.	.	.	.	.	.	.	T	T	.	.	C	.	G	.	A	.	G	.	.	A	.	-	-	-	-	A	C	.	.	.	.	.	.	.	.	.	.	.	.	.	.	.	.	.	C
AG 51H	.	.	.	.	.	.	.	.	.	.	.	T	.	.	.	C	.	G	.	A	.	G	.	.	A	.	-	-	-	-	A	C	.	.	.	.	.	.	.	.	.	.	.	.	.	G	.	.	.	C
GAL 5M	.	.	.	.	.	.	.	.	.	.	.	.	.	.	A	C	.	G	.	.	T	G	.	.	A	.	-	-	-	-	A	.	.	.	.	.	.	.	.	.	.	C	C	.	.	.	.	.	.	.
GAL 16M	G	.	.	.	.	.	.	.	.	.	.	.	.	.	.	C	.	G	.	.	.	G	.	.	A	.	-	-	-	-	A	.	.	.	.	.	.	.	.	.	.	C	C	.	.	.	.	.	G	.
GAL 23M	.	.	.	.	.	.	.	.	.	.	.	.	.	.	.	C	.	G	.	.	T	G	.	.	A	.	-	-	-	-	A	.	.	.	.	.	.	.	.	.	.	C	C	.	.	.	.	.	.	.
GAL 24M	G	.	.	.	.	.	.	.	.	.	.	.	.	.	.	C	.	G	.	.	.	G	.	.	A	.	-	-	-	-	A	.	.	.	.	.	.	.	.	.	.	C	C	.	.	.	.	.	G	.
GAL 2H	.	.	.	.	.	.	.	.	.	.	C	.	.	.	A	C	.	G	.	.	.	G	.	.	A	.	-	-	-	-	A	.	.	.	.	.	A	.	.	.	.	C	C	.	.	.	.	.	.	.
GAL 16H	G	.	.	.	.	.	.	.	.	.	.	.	.	.	.	C	.	G	.	.	.	G	.	.	A	.	-	-	-	-	A	.	.	.	.	.	.	.	.	.	.	C	C	.	.	.	.	.	.	.
GAL 21H	.	.	.	.	.	.	.	.	.	.	C	.	.	.	A	C	.	G	.	.	.	G	.	.	A	.	-	-	-	-	A	.	.	.	.	.	.	.	.	.	.	C	C	.	.	.	.	.	.	.
GAL 26H	.	.	.	.	.	.	.	.	.	.	C	.	.	.	A	C	.	G	.	.	.	G	.	.	A	.	-	-	-	-	A	.	.	.	.	.	.	.	.	.	.	C	C	.	.	.	.	.	.	.
GAL 27H	.	G	.	.	.	.	.	.	.	.	.	.	.	.	.	C	.	G	.	.	.	G	.	.	A	.	-	-	-	-	A	.	.	.	.	.	.	.	.	.	.	C	C	.	.	.	.	.	.	.
GAL 30H	G	.	.	.	.	.	.	.	.	.	.	.	.	.	.	C	.	G	.	.	.	G	.	.	A	.	-	-	-	-	A	.	.	.	.	.	.	.	.	.	.	C	C	.	.	.	.	.	G	.
AK 3M	.	.	.	.	.	.	.	.	.	.	.	T	.	.	.	C	.	G	.	.	.	G	A	.	A	.	G	T	G	C	A	.	.	.	.	.	.	.	.	.	.	.	.	.	.	.	.	.	.	.
AK 5M	.	.	.	C	.	.	.	.	.	.	.	T	.	.	.	C	.	G	.	.	.	G	.	.	A	.	-	-	-	-	A	.	.	.	.	.	.	T	.	.	.	.	.	.	.	.	.	.	.	.
AK 18M	.	.	.	.	.	.	.	.	.	.	.	T	.	.	.	C	.	G	.	.	.	G	A	.	A	.	G	C	G	C	A	.	.	.	.	.	.	.	.	.	.	.	.	A	.	.	A	T	.	.
AK 20M	.	.	A	.	.	.	.	.	-	-	-	-	-	-	-	-	-	-	-	-	-	-	-	-	-	-	-	-	-	-	-	-	-	-	-	-	-	-	-	.	.	.	.	.	.	.	.	.	.	.
AK 21M	.	.	.	.	.	.	.	.	.	.	.	T	.	.	.	C	.	G	.	.	.	G	A	.	A	.	G	T	G	C	A	.	.	.	.	.	.	.	.	.	.	.	.	.	.	T	.	.	.	.
AK 24M	.	.	.	.	.	.	.	.	.	.	.	T	.	.	.	C	.	G	.	.	.	G	A	.	A	.	A	C	G	C	A	.	.	.	.	.	.	.	.	.	.	.	.	.	.	.	.	.	.	.
AK 2H	.	.	.	.	.	.	.	.	-	-	-	-	-	-	-	-	-	-	-	-	-	-	-	-	-	-	-	-	-	-	-	-	-	-	-	-	-	-	-	.	.	.	.	G	.	.	.	.	.	.
AK 21H	.	.	.	.	.	.	.	.	A	.	.	T	.	.	.	C	.	G	.	.	.	G	.	.	A	.	-	-	-	-	A	.	.	.	.	.	.	.	.	.	T	.	.	.	.	.	.	.	.	.
AK 23H	.	.	.	.	.	T	.	.	.	.	.	T	.	.	.	C	.	G	.	.	.	G	A	.	A	.	G	C	T	T	A	.	.	T	.	.	.	.	.	.	.	.	.	.	.	.	.	.	.	.
AK 26H	.	.	.	.	.	.	.	.	.	A	.	T	.	.	.	C	.	G	.	.	.	G	.	.	A	.	-	-	-	-	A	.	.	.	.	.	.	.	T	.	.	.	.	.	.	.	.	.	.	.
AK 52H	.	.	.	.	.	.	.	.	-	-	-	-	-	-	-	-	-	-	-	-	-	-	-	-	-	-	-	-	-	-	-	-	-	-	-	-	-	-	-	.	.	.	.	G	.	.	.	.	.	.
AH2 9M	.	.	.	.	.	.	A	.	.	.	.	T	.	.	.	C	.	G	.	.	.	G	.	.	A	.	-	-	-	-	A	.	.	.	.	.	.	.	.	T	.	.	.	.	A	.	.	.	.	.
AH2 17M	.	.	.	.	.	.	A	.	.	.	.	T	.	.	.	C	.	G	.	.	.	G	.	.	A	.	-	-	-	-	A	.	.	.	.	.	.	.	.	T	.	.	.	.	.	.	.	.	.	.
AH2 31H	.	.	.	.	.	.	.	.	.	.	.	T	.	.	.	C	.	G	.	.	.	G	.	.	A	.	-	-	-	-	A	.	.	.	.	.	.	.	.	.	.	.	.	.	.	.	.	.	.	.
AH2 50H	.	.	.	.	.	.	.	.	.	.	.	T	.	.	.	C	.	G	.	.	.	G	.	.	A	.	-	-	-	-	A	.	.	.	.	.	.	.	.	.	.	.	.	.	.	.	.	.	.	.
AH2 52H	.	.	.	.	.	.	A	.	.	.	.	T	.	.	.	C	.	G	.	.	.	G	.	.	A	.	-	-	-	-	A	.	.	.	.	.	.	.	.	T	.	.	.	.	A	.	.	.	.	.

Horizontal lines separate the A sequences included in the different breakpoint regions (AB, AG, GAL, AK and AH2). A dash indicates a nucleotide deletion and a question mark a non-sequenced site. Grey shadowed sites in a particular group of sequences highlight those sites not analyzed in that group in the complete-deletion option. Boxes group polymorphic sites either affected by the same deletion or not sequenced. Sites with multiple hits are indicated by red numbers when considering all sequences and by red letters when within a particular group of sequences. Horizontal lines above polymorphic sites indicate sites located within homologous sequences of snoRNA genes.

Supplementary Table S2. Nucleotide polymorphisms in the 4243-nt long multiple alignment of fragment A from five different breakpoint regions.

																																	Dpse\snoRNA: GA29821																	
	3600	3603	3607	3610	3613	3617	3620	3621	3629	3630	3632	3633	3642	3648	3650	3651	3661	3663	3672	3681	3697	3698	3699	3703	3704	3706	3709	3711	3712	3715	3725	3727	3730	3734	3746	3769	3777	3779	3787	3803	3817	3818	3827	3837	3843	3852	3854	3865	3866	3873
AB 4M	T	C	A	G	C	C	G	G	A	A	G	C	A	T	C	A	C	C	T	T	T	T	T	A	G	A	G	A	A	A	A	A	A	G	T	C	T	T	T	A	A	G	G	T	C	G	T	A	C	G
AB 5M	.	.	.	.	.	.	.	.	.	.	.	T	.	.	.	.	T	.	.	G	.	G	.	.	.	.	.	.	.	.	.	.	.	.	.	.	.	.	G	T	T	.	.	.	.	.	.	.	.	.
AB 7M	.	.	.	.	.	.	.	.	.	.	.	.	.	.	.	.	T	.	.	G	.	G	.	.	.	.	.	.	.	.	.	G	.	.	.	.	.	.	G	.	.	.	.	.	.	.	.	.	.	.
AB 14M	.	.	.	.	.	.	.	.	.	.	.	.	.	.	.	.	T	.	.	G	.	G	.	G	.	.	.	.	G	.	.	.	.	.	.	.	.	.	.	.	T	.	.	.	.	.	G	.	.	.
AB 16M	.	.	.	.	.	.	.	.	.	G	.	.	.	.	.	.	T	.	.	G	.	G	.	.	.	.	.	.	.	.	.	.	.	.	.	.	.	.	G	.	.	.	.	.	.	.	.	G	.	.
AB 17M	.	.	.	.	.	.	.	.	.	.	.	.	.	.	.	.	T	.	.	G	.	G	.	.	.	.	.	.	.	.	.	.	.	.	.	.	G	.	G	.	.	C	.	.	.	C	.	.	.	.
AB 23M	.	.	.	.	.	.	.	.	.	.	.	.	.	.	.	.	T	.	.	G	.	G	.	.	.	.	.	.	.	.	.	G	.	.	.	.	.	.	G	.	.	.	.	.	.	.	.	.	.	.
AB 25M	.	.	.	.	.	.	.	.	.	.	.	.	.	.	.	.	T	.	.	G	.	G	.	.	.	.	.	.	.	.	.	.	.	.	.	.	G	.	G	.	.	C	.	.	.	C	.	.	.	.
AB 2H	.	.	.	.	.	.	.	.	.	T	.	.	.	.	.	.	T	.	.	G	.	G	.	G	.	.	.	.	G	.	.	.	.	.	.	.	.	.	G	.	T	.	.	.	.	.	G	.	.	.
AB 11H	.	.	.	.	.	.	.	.	.	.	.	.	.	.	.	.	T	.	.	G	.	G	.	.	.	.	.	.	.	.	.	.	.	.	.	.	G	.	G	.	.	C	.	.	.	C	.	.	.	.
AB 16H	.	.	.	.	A	.	.	.	.	.	.	.	.	.	.	.	T	.	.	G	.	G	.	.	.	.	.	.	.	G	.	.	T	.	.	.	.	.	G	.	.	.	.	.	.	.	.	.	.	.
AB 17H	.	.	.	.	.	.	.	.	.	.	.	.	.	.	.	.	T	.	.	G	.	G	.	.	.	.	.	.	.	.	.	.	.	.	.	.	.	.	G	.	T	.	.	.	.	.	.	.	.	.
AB 18H	.	.	.	.	.	.	.	.	.	.	.	.	.	.	.	.	T	.	.	G	.	G	.	.	.	.	.	.	.	G	.	.	T	.	.	.	.	.	G	.	.	.	.	.	.	.	.	.	.	.
AB 21H	.	.	.	.	.	.	.	.	.	.	.	.	.	.	.	.	.	.	.	.	.	.	.	.	.	.	.	.	.	.	.	.	.	.	.	.	.	.	.	.	.	.	.	.	.	.	.	.	.	.
AB 26H	.	.	.	.	.	.	.	.	.	.	.	.	.	.	.	.	T	.	.	G	.	G	.	.	.	.	.	.	.	.	.	.	.	.	.	.	.	.	G	.	T	.	.	.	.	.	.	.	.	.
AB 27H	.	.	.	.	.	.	.	.	.	G	.	.	.	.	.	.	T	.	.	G	.	G	.	.	.	.	.	.	.	.	.	.	.	.	.	.	.	.	G	.	T	.	.	.	.	.	.	.	.	.
AB 30H	.	.	.	.	.	.	.	.	.	.	.	.	.	.	.	.	.	.	.	.	.	.	.	.	.	.	.	.	.	.	.	.	.	.	.	.	.	.	.	.	.	.	.	.	.	.	.	.	.	.
AB 51H	.	.	.	.	.	.	.	.	.	.	.	.	.	.	.	.	T	.	.	G	.	G	.	.	.	.	.	.	.	.	.	.	.	.	.	.	.	.	G	.	T	.	.	.	.	.	.	.	.	.
AG 4M	.	.	C	A	.	T	.	.	.	.	.	.	.	.	.	.	T	.	.	G	.	G	.	.	A	.	C	G	.	.	-	T	.	.	.	.	.	.	G	.	.	.	.	.	.	.	.	.	T	.
AG 7M	.	.	C	A	.	T	.	.	.	.	.	.	.	.	.	.	T	.	.	G	.	G	.	.	A	.	C	G	.	.	-	T	.	.	.	.	.	.	G	.	.	.	.	.	.	.	.	.	.	.
AG 25M	.	.	C	A	.	T	.	.	.	.	.	.	.	.	.	.	T	.	.	G	.	G	.	.	A	.	C	G	.	.	-	T	.	.	.	.	.	.	G	.	.	.	.	.	.	.	.	.	.	.
AG 17H	.	.	C	A	.	T	.	.	.	.	.	.	.	.	.	.	T	.	.	G	.	G	.	.	A	.	C	G	.	.	-	T	.	.	.	.	.	.	G	.	.	.	.	.	.	.	.	.	.	.
AG 25H	.	.	C	A	.	T	.	.	.	.	.	.	.	.	.	.	T	.	.	G	.	G	.	.	A	.	C	G	.	.	-	T	.	.	.	.	.	.	G	.	.	.	.	.	.	.	.	.	.	.
AG 51H	.	.	C	A	.	T	.	.	.	.	.	.	.	.	.	.	T	.	.	G	.	.	G	.	A	.	C	.	.	.	T	T	.	.	.	.	.	.	G	.	.	.	.	.	.	.	.	.	.	.
GAL 5M	.	.	C	A	.	T	.	.	G	.	.	.	.	.	.	.	T	.	C	G	C	G	.	.	A	.	C	.	.	.	-	T	.	.	.	.	.	.	G	.	.	.	.	.	.	.	.	.	.	A
GAL 16M	.	.	C	A	.	T	.	.	G	.	.	.	G	.	T	T	T	.	C	G	.	G	.	.	A	.	C	.	.	.	-	T	.	.	.	.	.	.	G	.	.	.	.	.	T	.	.	.	.	A
GAL 23M	.	.	C	A	.	T	.	.	G	.	.	.	G	.	.	.	T	.	C	G	.	G	.	.	A	.	C	.	.	.	-	T	.	.	.	.	.	.	G	.	.	.	.	.	.	.	.	.	.	A
GAL 24M	.	.	C	A	.	T	.	.	G	.	.	.	G	.	.	.	T	.	C	G	.	G	.	.	A	.	C	.	.	.	-	T	.	.	.	.	.	.	G	.	.	.	.	.	T	.	.	.	.	A
GAL 2H	.	.	C	A	.	T	.	.	G	.	.	.	.	.	.	.	T	.	C	G	.	G	.	.	A	.	C	.	.	.	-	T	.	.	.	.	.	.	G	.	.	.	.	.	.	.	.	.	.	A
GAL 16H	.	.	C	A	.	T	.	.	G	.	.	.	G	.	.	.	T	.	C	G	.	G	.	.	A	.	C	.	.	.	-	T	.	.	.	.	.	.	G	.	.	.	.	.	.	.	.	.	.	A
GAL 21H	.	.	C	A	.	T	.	.	G	.	.	.	.	.	.	.	T	.	C	G	.	G	.	.	A	.	C	.	.	.	-	T	.	.	.	.	.	.	G	.	.	.	.	.	.	.	.	.	.	A
GAL 26H	.	.	C	A	.	T	.	.	G	.	.	.	.	.	.	.	T	.	C	G	.	G	.	.	A	.	C	.	.	.	-	T	.	.	.	.	.	.	G	.	.	.	.	.	.	.	.	.	.	A
GAL 27H	.	.	C	A	.	T	T	.	G	.	.	.	.	.	.	.	T	.	C	G	.	G	.	.	A	.	C	.	.	.	-	T	.	.	.	.	.	.	G	.	.	.	.	.	.	.	.	.	.	A
GAL 30H	.	.	C	A	.	T	.	.	G	.	.	.	G	.	.	.	T	.	C	G	.	G	.	.	A	.	C	.	.	.	-	T	.	.	.	.	.	.	G	.	.	.	.	.	T	.	.	.	.	A
AK 3M	.	.	.	.	.	.	.	A	.	.	.	.	.	.	.	.	T	.	.	G	.	G	.	.	.	.	.	.	.	.	.	.	.	A	?	?	?	?	?	?	?	?	?	?	?	?	?	?	?	?
AK 5M	.	.	.	.	.	.	.	.	.	.	.	.	.	.	.	.	T	.	-	G	.	G	.	.	.	G	.	.	.	.	.	.	.	.	.	.	.	C	G	.	.	.	.	.	.	.	.	.	.	.
AK 18M	.	.	.	.	.	.	.	.	.	.	.	.	.	.	.	.	T	.	.	G	.	G	.	.	.	.	.	.	.	.	.	.	.	A	?	?	?	?	?	?	?	?	?	?	?	?	?	?	?	?
AK 20M	.	.	.	.	.	.	.	.	.	.	A	.	.	.	.	.	T	.	.	G	.	G	.	.	.	.	.	.	.	.	.	.	.	.	.	.	.	C	G	.	.	.	.	.	.	.	.	.	.	.
AK 21M	C	.	.	.	.	.	.	.	.	.	.	.	.	.	.	.	T	.	.	G	.	G	.	.	.	.	.	.	.	.	.	.	.	A	?	?	?	?	?	?	?	?	?	?	?	?	?	?	?	?
AK 24M	.	.	.	.	.	.	.	.	.	.	.	.	.	.	.	.	T	.	.	G	.	G	.	.	.	.	.	.	.	.	.	.	.	A	?	?	?	?	?	?	?	?	?	?	?	?	?	?	?	?
AK 2H	.	.	.	.	.	.	.	.	.	.	A	.	.	.	.	.	T	.	.	G	.	G	.	.	.	.	.	.	.	.	.	.	.	.	.	.	.	C	G	.	.	.	.	.	.	.	.	.	.	.
AK 21H	.	.	.	.	.	.	.	.	.	.	.	.	.	A	.	.	T	A	.	G	.	G	.	.	.	.	.	.	.	.	.	.	.	.	.	G	.	C	G	.	.	.	.	.	.	.	.	.	.	.
AK 23H	.	.	.	.	.	.	.	.	.	.	.	.	.	.	.	.	T	.	.	G	.	G	.	.	.	.	.	.	.	.	.	.	.	A	?	?	?	?	?	?	?	?	?	?	?	?	?	?	?	?
AK 26H	.	T	.	.	.	.	.	.	.	.	.	.	.	A	.	.	T	A	.	G	.	G	.	.	.	.	.	.	.	.	.	.	.	.	.	.	.	C	G	.	.	.	.	.	.	.	.	.	.	.
AK 52H	.	.	.	.	.	.	.	.	.	.	A	.	.	.	.	.	T	.	.	G	.	G	.	.	.	.	.	.	.	.	.	.	.	.	.	.	.	C	G	.	.	.	A	.	.	.	.	.	.	.
AH2 9M	.	.	.	.	.	.	.	.	.	.	.	.	.	.	.	.	T	.	.	G	.	G	.	.	.	.	.	.	.	.	.	.	.	.	G	.	.	C	G	.	.	.	.	A	A	.	.	.	.	.
AH2 17M	.	.	.	.	.	.	.	.	.	.	.	.	.	.	.	.	T	.	.	G	.	G	.	.	.	.	.	.	.	.	.	.	.	.	G	.	.	C	G	.	.	.	.	A	A	.	.	.	.	.
AH2 31H	.	.	.	.	.	.	.	.	.	.	.	.	.	.	.	.	T	.	.	G	.	G	.	.	.	.	.	.	.	.	.	.	.	.	G	.	.	C	G	.	.	.	.	A	A	.	.	.	.	.
AH2 50H	.	.	.	.	.	.	.	.	.	.	.	.	.	.	.	.	T	.	.	G	.	G	.	.	.	.	.	.	.	.	.	.	.	.	G	.	.	C	G	.	.	.	.	A	A	.	.	.	.	.
AH2 52H	.	.	.	.	.	.	.	.	.	.	.	.	.	.	.	.	T	.	.	G	.	G	.	.	.	.	.	.	.	.	.	.	.	.	G	.	.	C	G	.	.	.	.	A	A	.	.	.	.	.

Horizontal lines separate the A sequences included in the different breakpoint regions (AB, AG, GAL, AK and AH2). A dash indicates a nucleotide deletion and a question mark a non-sequenced site. Grey shadowed sites in a particular group of sequences highlight those sites not analyzed in that group in the complete-deletion option. Boxes group polymorphic sites either affected by the same deletion or not sequenced. Sites with multiple hits are indicated by red numbers when considering all sequences and by red letters when within a particular group of sequences. Horizontal lines above polymorphic sites indicate sites located within homologous sequences of snoRNA genes.

(continued)

Supplementary Table S2. Nucleotide polymorphisms in the 4243-nt long multiple alignment of fragment A from five different breakpoint regions.

	3876	3877	3883	3894	3900	3904	3909	3915	3916	3928	3931	3932	3938	3943	3950	3956	3958	3963	3969	3971	3990	3991	4002	4015	4017	4018	4019	4021	4049	4058	4063	4065	4067	4068	4070	4071	4077	4079	4080	4086	4098	4099	4105	4112	4125	4131	4133	4148	4151	4154
															Dpse\snoRNA: GA29820																																			
AB 4M	A	A	T	G	G	A	A	T	G	A	C	A	G	A	T	T	G	C	T	T	A	T	T	T	T	G	T	C	C	A	T	A	A	A	T	A	A	C	T	C	-	A	C	C	A	A	G	A	G	C
AB 5M	.	.	.	.	.	.	.	.	.	.	T	.	.	T	.	A	T	T	.	.	.	.	.	A	.	.	.	.	.	.	.	.	.	.	.	G	.	.	.	.	-	.	.	.	.	.	.	.	.	.
AB 7M	G	.	.	C	.	G	.	.	.	.	.	.	.	.	.	.	.	.	.	.	.	.	.	A	.	.	.	.	.	.	.	.	.	.	.	G	.	.	.	.	C	.	.	.	.	.	.	.	.	T
AB 14M	.	.	.	.	.	.	.	.	.	.	.	.	.	.	.	.	.	.	.	.	.	.	.	A	.	.	.	.	.	.	.	.	.	.	.	-	.	.	.	.	-	.	.	.	.	.	.	T	.	.
AB 16M	.	.	.	.	.	.	.	.	.	.	.	.	.	.	.	.	.	.	.	.	.	.	.	.	.	.	.	.	.	.	.	.	.	.	.	.	.	.	.	.	-	.	.	.	.	.	.	.	.	.
AB 17M	G	.	.	C	.	G	.	G	T	.	.	.	.	.	.	.	.	.	.	.	.	.	.	.	.	.	.	.	.	.	.	.	.	.	.	-	.	.	.	.	-	.	.	.	.	.	.	T	.	.
AB 23M	G	.	.	C	.	G	.	.	.	.	.	.	.	.	.	.	.	.	.	.	.	.	.	A	.	.	.	.	.	.	.	.	.	.	.	G	.	.	.	.	C	.	.	.	.	.	.	.	.	T
AB 25M	G	.	.	C	.	G	.	G	T	.	.	.	.	.	.	.	.	.	.	.	.	.	.	.	.	.	.	.	.	.	.	.	.	.	.	-	.	.	.	.	-	.	.	.	.	.	.	T	.	.
AB 2H	.	.	.	.	.	.	.	.	.	.	.	.	.	.	.	.	.	.	.	.	.	.	.	A	.	.	.	.	T	.	.	.	.	.	.	G	.	.	.	.	-	.	.	.	.	.	.	.	.	.
AB 11H	G	.	.	C	.	G	.	G	T	.	.	.	.	.	.	.	.	.	.	.	.	.	.	.	.	.	.	.	.	.	.	.	.	.	.	-	.	.	.	.	-	.	.	.	.	.	.	T	.	.
AB 16H	.	.	.	.	.	.	.	.	.	.	.	.	.	.	.	.	.	.	.	.	.	.	.	.	.	.	.	.	.	.	.	.	.	.	.	.	.	.	.	.	-	.	.	.	.	.	.	.	.	.
AB 17H	.	.	.	.	.	.	.	.	.	.	.	.	.	.	.	.	.	.	.	.	.	.	.	.	.	.	.	.	.	.	.	.	.	.	.	.	.	.	.	.	-	.	.	.	.	.	.	.	.	.
AB 18H	.	.	.	.	.	.	.	.	.	.	.	.	.	.	.	.	.	.	.	.	.	.	.	.	.	.	.	T	.	.	.	.	.	.	.	.	.	.	.	.	-	.	.	.	.	.	.	.	.	.
AB 21H	.	G	.	.	.	.	.	.	.	.	.	.	.	.	.	.	.	.	.	.	.	.	.	.	.	.	.	.	.	.	.	.	.	.	.	.	.	.	.	.	-	.	.	.	.	.	.	.	.	.
AB 26H	.	.	.	.	.	.	.	.	.	.	.	.	.	T	.	A	T	T	.	.	.	.	.	A	.	.	.	.	T	.	.	.	.	.	.	G	.	.	.	.	-	.	.	.	.	.	.	.	.	.
AB 27H	.	.	.	.	.	.	.	.	.	.	.	.	.	T	.	A	T	T	C	.	.	.	.	A	.	.	.	.	.	.	.	.	.	.	.	G	.	.	.	.	-	.	.	.	.	.	.	.	.	.
AB 30H	.	.	.	.	.	.	.	.	.	.	.	.	.	.	.	.	.	.	.	.	.	.	.	.	.	.	.	.	.	.	.	.	.	.	.	.	.	.	.	.	-	.	.	.	.	.	.	.	.	.
AB 51H	.	.	.	.	.	.	.	.	.	.	.	.	.	T	.	A	T	T	.	.	.	.	.	A	.	.	.	.	T	.	.	.	.	.	.	G	.	.	.	.	-	.	.	.	.	.	.	.	.	.
AG 4M	G	.	.	.	.	.	.	.	.	.	.	.	.	.	.	.	.	T	.	.	.	.	.	.	.	.	.	.	.	.	.	.	.	.	A	.	.	.	.	.	-	.	.	T	.	T	.	.	T	.
AG 7M	G	.	.	.	.	.	.	.	.	.	.	.	.	.	.	.	.	T	.	.	.	.	.	.	.	.	.	.	.	.	.	.	.	.	A	.	.	.	.	.	-	.	.	.	.	T	.	.	T	.
AG 25M	G	.	.	.	.	.	.	.	.	.	.	.	.	.	.	.	.	T	.	.	.	.	.	.	.	.	.	.	.	.	.	.	.	.	A	.	.	.	.	.	-	.	.	.	.	T	.	.	T	.
AG 17H	G	.	.	.	.	.	.	.	.	.	.	.	.	.	.	.	.	T	.	.	.	.	.	.	.	.	.	.	.	.	.	.	.	.	A	.	.	.	.	.	-	.	.	.	.	T	.	.	T	.
AG 25H	G	.	.	.	.	.	.	.	.	.	.	.	.	.	.	.	.	T	.	.	.	.	.	.	.	.	.	.	.	.	.	.	.	.	A	.	.	.	.	.	-	.	.	.	.	T	.	.	T	.
AG 51H	G	.	.	C	.	.	.	.	.	.	.	.	.	.	t	.	.	T	.	.	.	.	.	.	.	.	.	.	.	.	.	.	.	.	.	.	.	.	.	.	T	G	.	T	C	.	A	.	.	.
GAL 5M	G	.	.	C	.	.	.	.	.	.	.	.	C	.	A	.	.	T	.	.	.	.	.	.	.	.	.	.	.	.	.	.	.	.	.	.	.	A	.	A	-	.	.	.	.	.	.	.	.	.
GAL 16M	G	.	.	C	.	.	.	G	T	.	.	.	.	.	.	.	.	T	.	.	.	.	.	.	.	.	.	.	.	.	.	.	.	.	.	.	C	A	.	A	-	.	.	.	.	.	.	.	.	.
GAL 23M	G	.	.	C	.	.	.	G	T	.	.	.	.	.	.	.	.	T	.	.	G	.	.	.	.	.	.	.	.	.	.	.	.	.	.	.	C	A	.	A	-	.	.	.	.	.	.	.	.	.
GAL 24M	G	.	.	C	.	.	.	G	T	.	.	.	.	.	.	.	.	T	.	.	.	.	.	.	.	.	.	.	.	.	.	.	.	.	.	.	C	A	.	A	-	.	.	.	.	.	.	.	.	.
GAL 2H	G	.	.	C	.	.	.	.	.	.	.	.	C	.	A	.	.	T	.	.	a	.	.	.	.	.	.	.	.	.	.	.	.	.	.	.	.	A	.	A	-	.	.	.	.	.	.	.	.	.
GAL 16H	G	.	A	C	.	.	.	G	A	.	.	.	.	.	.	.	.	T	.	.	.	.	.	.	.	.	.	.	.	.	.	C	.	.	.	.	C	A	.	A	-	.	.	.	.	.	.	.	.	.
GAL 21H	G	.	.	C	.	.	.	G	.	.	.	G	.	.	.	.	.	T	.	.	.	.	.	.	.	.	.	.	.	T	.	.	.	.	.	.	.	.	.	.	T	G	.	.	.	.	.	.	.	.
GAL 26H	G	.	.	C	T	.	.	G	.	.	.	G	.	.	.	.	.	T	.	.	.	.	.	.	.	.	.	.	.	T	.	.	.	.	.	.	.	.	.	.	T	G	.	.	.	.	.	.	.	.
GAL 27H	G	.	.	C	.	.	.	G	.	.	.	G	.	.	.	.	.	T	.	.	.	.	.	.	G	.	.	.	.	.	.	.	.	.	.	.	.	.	G	.	T	G	.	.	.	.	.	.	.	.
GAL 30H	G	.	.	C	.	.	.	G	T	.	.	.	.	.	.	.	.	T	.	.	.	.	.	.	.	.	.	.	.	.	.	.	.	.	.	.	C	A	.	A	-	.	.	.	.	.	.	.	.	.
AK 3M	?	?	?	?	?	?	?	?	?	?	?	?	?	?	?	?	?	?	?	?	?	?	?	?	?	?	?	?	?	?	?	?	?	?	?	?	?	?	?	?	?	?	?	?	?	?	?	?	?	?
AK 5M	G	.	.	C	.	.	.	G	.	.	.	.	.	.	.	.	.	.	.	.	.	.	C	A	.	T	.	.	.	.	C	.	T	T	.	.	.	.	.	.	-	.	.	.	.	.	A	.	.	.
AK 18M	?	?	?	?	?	?	?	?	?	?	?	?	?	?	?	?	?	?	?	?	?	?	?	?	?	?	?	?	?	?	?	?	?	?	?	?	?	?	?	?	?	?	?	?	?	?	?	?	?	?
AK 20M	G	.	.	C	.	.	.	G	.	.	.	.	.	.	.	.	.	.	.	.	.	.	.	A	A	T	.	.	.	.	C	.	T	T	.	.	.	.	.	.	-	.	.	.	.	.	.	.	.	.
AK 21M	?	?	?	?	?	?	?	?	?	?	?	?	?	?	?	?	?	?	?	?	?	?	?	?	?	?	?	?	?	?	?	?	?	?	?	?	?	?	?	?	?	?	?	?	?	?	?	?	?	?
AK 24M	?	?	?	?	?	?	?	?	?	?	?	?	?	?	?	?	?	?	?	?	?	?	?	?	?	?	?	?	?	?	?	?	?	?	?	?	?	?	?	?	?	?	?	?	?	?	?	?	?	?
AK 2H	G	.	.	C	.	.	.	G	.	.	.	.	.	.	.	.	.	.	.	C	.	.	.	A	.	T	.	.	.	.	C	.	T	T	.	.	.	.	.	.	-	.	.	.	.	.	.	.	.	.
AK 21H	G	.	.	C	.	.	.	G	.	.	.	.	.	.	.	.	.	.	.	.	.	.	C	A	.	T	.	.	.	.	C	.	T	T	.	.	.	.	.	.	-	.	.	.	.	.	.	.	.	.
AK 23H	?	?	?	?	?	?	?	?	?	?	?	?	?	?	?	?	?	?	?	?	?	?	?	?	?	?	?	?	?	?	?	?	?	?	?	?	?	?	?	?	?	?	?	?	?	?	?	?	?	?
AK 26H	G	.	.	C	.	.	.	G	.	.	.	.	.	.	.	.	.	.	.	.	.	.	C	A	.	T	.	.	.	.	C	.	T	T	.	.	.	.	.	.	-	.	.	.	.	.	A	.	.	.
AK 52H	G	.	.	A	.	.	G	G	.	C	.	.	.	.	.	.	.	.	.	.	.	A	.	A	.	.	.	.	.	.	C	.	T	T	.	.	.	.	.	.	-	.	.	.	.	.	A	.	.	.
AH2 9M	G	.	.	C	.	.	.	G	.	.	.	.	.	.	.	.	.	.	.	.	.	.	.	A	.	.	C	.	.	.	C	.	T	T	.	.	.	.	.	.	-	.	.	.	.	.	.	.	.	.
AH2 17M	G	.	.	C	.	.	.	G	.	.	.	.	.	.	.	.	.	.	.	.	.	.	.	A	.	.	C	.	.	.	C	.	T	T	.	.	.	.	.	.	-	.	.	.	.	.	.	.	.	.
AH2 31H	G	.	.	C	.	.	.	G	.	.	.	.	.	.	.	.	.	.	.	.	.	.	.	A	.	.	C	.	.	.	C	.	T	T	.	.	.	.	.	.	-	.	G	.	.	.	.	.	.	.
AH2 50H	G	.	.	C	.	.	.	G	.	.	.	.	.	.	.	.	.	.	.	.	.	.	.	A	.	.	C	.	.	.	C	.	T	T	.	.	.	.	.	.	-	.	G	.	.	.	.	.	.	.
AH2 52H	G	.	.	C	.	.	.	G	.	.	.	.	.	.	.	.	.	.	.	.	.	.	.	A	.	.	C	.	.	.	C	.	T	T	.	.	.	.	.	.	-	.	.	.	.	.	.	.	.	.

Horizontal lines separate the A sequences included in the different breakpoint regions (AB, AG, GAL, AK and AH2). A dash indicates a nucleotide deletion and a question mark a non-sequenced site. Grey shadowed sites in a particular group of sequences highlight those sites not analyzed in that group in the complete-deletion option. Boxes group polymorphic sites either affected by the same deletion or not sequenced. Sites with multiple hits are indicated by red numbers when considering all sequences and by red letters when within a particular group of sequences. Horizontal lines above polymorphic sites indicate sites located within homologous sequences of snoRNA genes.

Supplementary Table S2. Nucleotide polymorphisms in the 4243-nt long multiple alignment of fragment A from five different breakpoint regions.

	4162	4169	4171	4188	4190	4192	4194	4197	4203	4217	4232
										Dpse\snoRNA: GA29819	
AB 4M	G	A	G	T	C	T	T	A	A	A	G
AB 5M	.	.	-	.	T	.	A	.	.	T	.
AB 7M	A	.	-	.	T	.	C	.	.	T	.
AB 14M	.	.	T	.	.	.	.	.	-	.	.
AB 16M	.	.	T	.	.	.	.	.	.	.	.
AB 17M	.	.	T	.	.	.	.	.	T	.	.
AB 23M	.	.	-	.	T	.	C	.	.	T	C
AB 25M	.	.	T	.	.	.	.	.	T	.	.
AB 2H	.	.	T	.	.	.	.	.	.	.	.
AB 11H	.	.	T	.	.	.	.	.	T	.	.
AB 16H	.	.	.	.	.	.	.	.	.	.	.
AB 17H	.	.	.	.	.	.	.	.	.	.	.
AB 18H	.	.	T	.	.	.	.	.	.	.	.
AB 21H	.	.	.	.	.	.	.	.	.	.	.
AB 26H	.	.	-	.	T	.	A	.	.	T	.
AB 27H	.	.	-	.	T	.	A	.	.	T	.
AB 30H	.	.	.	.	.	.	.	.	.	.	.
AB 51H	.	.	-	.	T	.	A	.	.	T	.
AG 4M	.	.	T	.	.	.	.	.	.	T	.
AG 7M	.	.	T	.	.	.	.	.	.	T	.
AG 25M	.	.	T	.	.	.	.	.	.	T	.
AG 17H	.	.	T	.	.	.	.	.	.	T	.
AG 25H	.	.	T	.	.	.	.	.	.	T	.
AG 51H	.	.	T	.	.	.	.	.	.	T	.
GAL 5M	.	.	T	.	.	.	.	.	.	T	.
GAL 16M	.	.	T	.	.	.	.	.	.	T	.
GAL 23M	.	.	T	.	.	.	.	.	.	T	.
GAL 24M	.	.	T	.	.	.	.	.	.	T	.
GAL 2H	.	.	T	.	.	.	.	.	.	T	.
GAL 16H	.	.	T	.	.	.	.	.	.	T	.
GAL 21H	.	.	T	.	.	.	.	.	.	T	.
GAL 26H	.	.	T	.	.	.	.	.	.	T	.
GAL 27H	.	.	T	.	.	.	.	.	.	T	.
GAL 30H	.	.	T	.	.	.	.	.	.	T	.
AK 3M	?	?	?	?	?	?	?	?	?	?	?
AK 5M	.	.	-	A	T	A	.	.	.	T	.
AK 18M	?	?	?	?	?	?	?	?	?	?	?
AK 20M	.	.	-	A	T	A	.	.	.	T	.
AK 21M	?	?	?	?	?	?	?	?	?	?	?
AK 24M	?	?	?	?	?	?	?	?	?	?	?
AK 2H	.	.	-	A	T	A	.	.	.	T	.
AK 21H	.	.	-	A	T	A	.	.	.	T	.
AK 23H	?	?	?	?	?	?	?	?	?	?	?
AK 26H	.	T	-	.	T	.	.	.	.	T	.
AK 52H	.	T	-	.	T	.	.	.	.	-	.
AH2 9M	.	.	-	.	T	.	.	.	.	T	.
AH2 17M	.	.	-	.	T	.	.	G	.	T	.
AH2 31H	.	.	-	.	T	.	.	.	.	T	.
AH2 50H	.	.	-	.	T	.	.	.	.	T	.
AH2 52H	.	.	-	.	T	.	.	.	.	T	.

Horizontal lines separate the A sequences included in the different breakpoint regions (AB, AG, GAL, AK and AH2). A dash indicates a nucleotide deletion and a question mark a non-sequenced site. Grey shadowed sites in a particular group of sequences highlight those sites not analyzed in that group in the complete-deletion option. Boxes group polymorphic sites either affected by the same deletion or not sequenced. Sites with multiple hits are indicated by red numbers when considering all sequences and by red letters when within a particular group of sequences. Horizontal lines above polymorphic sites indicate sites located within homologous sequences of snoRNA genes.

Supplementary Table S3. Primer pairs used to amplify fragment A in the different chromosomal arrangements.

Region	Forward oligonucleotide	Reverse oligonucleotide	size (Kb)	T (°C)	Time (min)
AB	GTTTCGCAGTCCCCAATGAG	CATTAGCGGGCAACGAAAAG	5.4	60	4
AG[a]	AGCTTGAGACGCCGACAGAC	ACAATGCACCCGTTGATTAGC	6.0	60	4
AK	AACCGCGACCATGTGCTAA	GCACACCGCAACATCACAC	6.6	59	4
AH2	AGAAACTAAGCCCAATAACTGA	AGAACTGCCCGTGCTAAA	6.6	59	4

[a] These primers amplify the A fragment of both the AG and GAL regions due to the duplication of section A_d (Figure 2 and text) during the E_9 inversion origin.

Supplementary Figure S1. Neighbor-joining tree of the A section sequences of homokatyotypic lines on different E chromosomal arrangements of *Drosophila subobscura*.

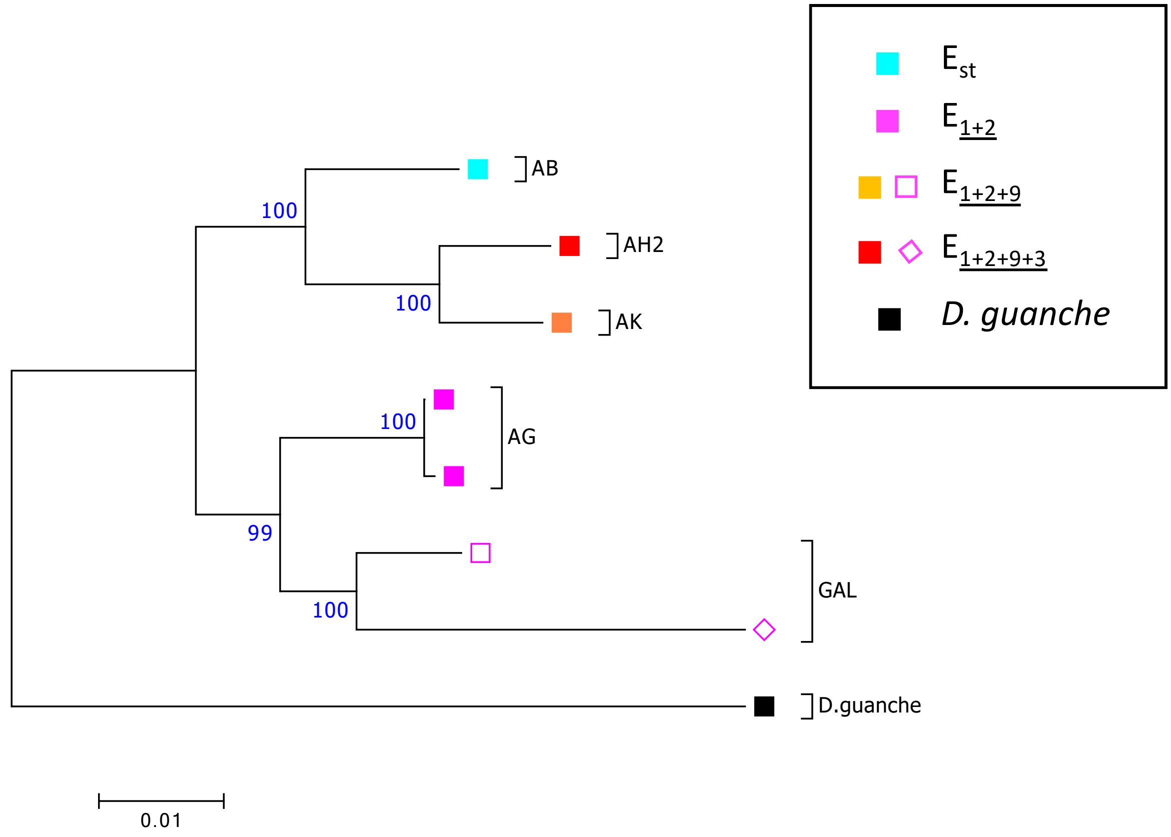

Bootstrap values (based on 1000 replicates) are shown on the tree. Only positions with less than 5% alignment gaps, missing data, and ambiguous bases were considered. *D. guanche* was used as outgroup.
